# Supplementary material for: Intratumoral Distribution of Lactate and the Monocarboxylate Transporters 1 and 4 in Human Glioblastoma Multiforme and Their Relationships to Tumor Progression-Associated Markers
Source: Int J Mol Sci. 2020 Aug 29;21(17):6254. doi: 10.3390/ijms21176254 (PMC7504270; doi:10.3390/ijms21176254)
Supplement: Supplementary file 1 [file ijms-21-06254-s001.zip › Table1S.pdf]

Lactate concentration (millimole = mM) in the contrast enhancing part of the tumor ("tumor"), the edema without contrast enhancement ("edema"), the normal appearing white matter ("NAWM") and the contralateral hemisphere ("contralateral site")

symbol of probe (P)	tumor					edema					NAWM					contralateral site				
	voxel	lactate concentration (mM)	standard deviation	average lactate concentration (mM)	average standard deviation	voxel	lactate concentration (mM)	standard deviation	average lactate concentration (mM)	average standard deviation	voxel	lactate concentration (mM)	standard deviation	average lactate concentration (mM)	average standard deviation	voxel	lactate concentration (mM)	standard deviation	average lactate concentration (mM)	average standard deviation
**P11**	12;12	107.00	23.00	107.00	23.00	13;10<br>10;11<br>10;12	27.00<br>51.00<br>39.00	61.00<br>48.00<br>50.00	39.00	53.00	10;13<br>14;11<br>14;12	0.00<br>16.00<br>0.00	999.00<br>101.00<br>999.00	5.33	699.67	13;5<br>14;5	1.00<br>0.00	999.00<br>999.00	0.50	999.00
**P12**	18;8<br>19;7<br>19;8	110.00<br>133.00<br>152.00	26.00<br>26.00<br>18.00	131.67	23.33	19;10	65.00	22.00	65.00	22.00	17;8<br>18;6<br>20;10	17.00<br>0.00<br>0.00	110.00<br>999.00<br>999.00	5.67	702.67	19;13	0.00	999.00	0.00	999.00
**P15**	10;4<br>11;4<br>11;5	200.00<br>134.00<br>164.00	13.00<br>15.00<br>16.00	166.00	14.67	9;3<br>11;3<br>13;5	84.00<br>88.00<br>58.00	29.00<br>23.00<br>28.00	76.67	26.67	5;4<br>5;5<br>6;3	3.00<br>15.00<br>0.00	374.00<br>178.00<br>999.00	6.00	517.00	4;10	0.00	999.00	0.00	999.00
**P17**	10;7<br>12;7<br>12;8	151.00<br>152.00<br>110.00	10.00<br>8.00<br>8.00	137.67	8.67	10;10<br>7;10<br>8;9	62.00<br>59.00<br>64.00	12.00<br>16.00<br>15.00	61.67	14.33	5;7<br>5;9<br>6;7	30.00<br>0.00<br>5.00	75.00<br>999.00<br>445.00	11.67	506.33	11;3<br>12;3	0.00<br>0.00	999.00<br>999.00	0.00	999.00
**P30**	11;4<br>12;4<br>12;6	16.00<br>11.00<br>9.00	4.00<br>21.00<br>7.00	12.00	4.00	14;4<br>13;6	5.00<br>3.00	10.00<br>20.00	4.00	15.00	7;6<br>7;7<br>15;5	0.00<br>2.00<br>1.00	999.00<br>44.00<br>74.00	1.00	521.50	8;12<br>10;12<br>11;12	1.00<br>1.00<br>1.00	80.00<br>75.00<br>71.00	1.00	77.50
**P42**	14;6<br>16;7<br>16;8	6.00<br>6.00<br>7.00	11.00<br>6.00<br>12.00	6.33	9.67	13;6<br>13;7	4.00<br>3.00	18.00<br>27.00	3.50	22.50	10;6<br>11;4<br>11;8	0.00<br>0.00<br>1.00	999.00<br>999.00<br>37.00	0.33	678.33	12;11<br>12;12	0.00<br>0.00	999.00<br>999.00	0.00	999.00
**P43**	13;13<br>14;11	117.00<br>104.00	14.00<br>10.00	110.50	12.00	11;10<br>11;11	66.00<br>62.00	17.00<br>16.00	64.00	16.50	11;9<br>15;9	18.00<br>0.00	62.00<br>999.00	9.00	530.50	12;6<br>12;7	0.00<br>0.00	999.00<br>999.00	0.00	999.00
**P44**	11;5<br>12;5<br>12;6	345.00<br>430.00<br>155.00	7.00<br>24.00<br>18.00	310.00	16.33	8;5<br>12;7<br>13;5	161.00<br>144.00<br>243.00	16.00<br>24.00<br>18.00	182.67	19.33	8;7<br>13;7	24.00<br>12.00	116.00<br>228.00	18.00	172.00	9;11	0.00	999.00	0.00	999.00

Relative lactate amount in the contrast enhancing part of the tumor ("tumor") and the edema without contrast enhancement ("edema")

normal appearing white matter = 1

symbol of probe (P)	tumor	edema
**P11**	20.075	7.317
**P12**	23.222	11.464
**P15**	27.667	12.778
**P17**	11.797	5.284
**P30**	12.000	4.000
**P42**	19.182	10.606
**P43**	12.278	7.111
**P44**	17.222	10.148

Depending on the size of the tumor, up to 3 different voxel were included in the calculation
