# Supplementary material for: Intratumoral Distribution of Lactate and the Monocarboxylate Transporters 1 and 4 in Human Glioblastoma Multiforme and Their Relationships to Tumor Progression-Associated Markers
Source: Int J Mol Sci. 2020 Aug 29;21(17):6254. doi: 10.3390/ijms21176254 (PMC7504270; doi:10.3390/ijms21176254)
Supplement: Supplementary file 1 [file ijms-21-06254-s001.zip › Table3S.pdf]

Correlation of the relative gene expression of MCT1 and MCT4 with the different EMT, stemness and domancy markers

MCT1	MCT4	vimentin	GFAP	ß-catenin	KLF4	OCT4	Sox2	EPHA5	H2BK	IGFBP5
center + edge	-0.099	-0.158	-0.248	-0.002	-0.256	0.105	0.209	-0.046	-0.046	-0.221
center	-0.240	0.040	0.004	0.204	-0.283	0.197	0.224	0.044	-0.036	-0.111
edge	-0.028	-0.288	-0.281	0.022	-0.194	0.150	0.190	-0.093	-0.123	-0.201

MCT4	MCT1	vimentin	GFAP	ß-catenin	KLF4	OCT4	Sox2	EPHA5	H2BK	IGFBP5
center + edge	0.419	-0.142	0.040	-0.074	-0.211	-0.158	0.060	-0.123	0.248	0.213
center	0.526	-0.080	0.083	-0.072	-0.493	-0.157	0.210	-0.239	0.421	0.375
edge	0.357	-0.140	0.116	-0.015	0.073	-0.185	-0.147	0.045	0.367	0.338

Correlation of the relative gene expression of MCT1, MCT4 and the different EMT, stemness and dormancy markers with the lactate concentration evaluated by lactate-ELISA

MCT1	MCT1	MCT4	vimentin	GFAP	ß-catenin	KLF4	OCT4	Sox2	EPHA5	H2BK	IGFBP5
center + edge	0.784	0.152	-0.252	-0.587	-0.490	-0.531	-0.416	0.196	-0.129	-0.007	-0.448
center	0.673	0.018	-0.132	-0.078	-0.114	-0.324	0.031	0.512	0.125	-0.237	0.265
edge	0.732	-0.256	-0.464	-0.706	-0.600	-0.566	-0.758	-0.302	0.111	-0.076	-0.639
t-value	6.437			-3.693							
p-value	0.000			0.001							
