# Supplementary material for: Intratumoral Distribution of Lactate and the Monocarboxylate Transporters 1 and 4 in Human Glioblastoma Multiforme and Their Relationships to Tumor Progression-Associated Markers
Source: Int J Mol Sci. 2020 Aug 29;21(17):6254. doi: 10.3390/ijms21176254 (PMC7504270; doi:10.3390/ijms21176254)
Supplement: Supplementary file 1 [file ijms-21-06254-s001.zip › Table2S.pdf]

symbol of probe (P)	MCT1			MCT4			Vimentin			GFAP		
	ΔCt	2^-(ΔCt)	n-fold	ΔCt	2^-(ΔCt)	n-fold	ΔCt	2^-(ΔCt)	n-fold	ΔCt	Sym	n-fold
P04 - center	1.845	0.278	0.665	4.708	0.038	0.360	-3.691	12.914	0.209	-3.139	8.808	3.000
P04 - edge	2.435	0.185		6.181	0.014		-1.430	2.694		-4.724	26.428	
P07 - center	1.458	0.364	0.319	5.866	0.017	2.477	-3.524	11.501	1.441	-6.053	66.383	3.338
P07 - edge	3.107	0.116		4.558	0.042		-4.051	16.579		-7.792	221.590	
P11 - center	0.892	0.539	0.705	3.356	0.098	0.631	-4.835	28.534	0.717	-5.040	32.905	0.455
P11 - edge	1.397	0.380		4.020	0.062		-4.355	20.466		-3.904	14.967	
P15 - center	0.989	0.504	1.168	3.436	0.092	0.597	-3.575	11.916	0.213	-5.294	39.238	3.256
P15 - edge	0.765	0.588		4.180	0.055		-1.342	2.535		-6.997	127.755	
P16 - center	1.920	0.264	0.475	2.714	0.152	0.928	-2.845	7.186	1.624	0.111	0.926	3.555
P16 - edge	2.993	0.126		2.822	0.141		-3.545	11.673		-1.719	3.291	
P19 - center	3.561	0.085	1.022	3.479	0.090	1.244	-3.514	11.427	1.046	-5.689	51.601	2.528
P19 - edge	3.530	0.087		3.163	0.112		-3.580	11.956		-7.028	130.473	
P23 - center	1.512	0.351	0.366	3.233	0.106	0.883	-4.814	28.124	0.416	-5.820	56.475	5.185
P23 - edge	2.961	0.128		3.412	0.094		-3.550	11.709		-8.194	292.807	
P24 - center	2.506	0.176	0.608	4.257	0.052	1.987	-3.777	13.708	1.995	-5.605	48.681	1.332
P24 - edge	3.224	0.107		3.266	0.104		-4.773	27.341		-6.018	64.821	
P26 - center	2.015	0.247	0.935	6.183	0.014	0.683	-3.238	9.432	0.888	-4.236	18.839	0.844
P26 - edge	2.112	0.231		6.733	0.009		-3.066	8.372		-3.992	15.909	
P27 - center	1.928	0.263	1.118	5.575	0.021	1.644	-1.689	3.225	1.021	-4.032	16.354	0.999
P27 - edge	1.767	0.294		4.857	0.035		-1.719	3.292		-4.030	16.333	
P30 - center	2.208	0.216	1.287	2.883	0.136	0.766	-3.496	11.283	1.292	-3.108	8.624	0.519
P30 - edge	1.843	0.279		3.267	0.104		-3.866	14.583		-2.161	4.473	
P36 - center	2.020	0.247	0.990	2.180	0.221	0.224	-3.051	8.285	0.680	-1.659	3.158	2.247
P36 - edge	2.034	0.244		4.337	0.049		-2.494	5.633		-2.827	7.095	
P21 - center	1.634	0.322	0.766	4.512	0.044	0.654	-4.195	18.317	1.428	-7.975	251.631	1.452
P21 - edge	2.019	0.247		5.124	0.029		-4.709	26.150		-8.514	365.472	
P34 - center	2.653	0.159	0.419	1.898	0.268	0.258	-5.564	47.306	1.117	-6.573	95.197	3.101
P34 - edge	3.907	0.067		3.850	0.069		-5.724	52.848		-8.206	295.193	
P35 - center	1.342	0.394	0.962	4.081	0.059	3.410	-3.293	9.801	3.409	-3.246	9.488	5.266
P35 - edge	1.397	0.380		2.311	0.202		-5.062	33.412		-5.643	49.964	

symbol of probe (P)	ß-Catenin			KLF4			Oct4			Sox2		
	ΔCt	2^-(ΔCt)	n-fold	ΔCt	2^-(ΔCt)	n-fold	ΔCt	2^-(ΔCt)	n-fold	ΔCt	2^-(ΔCt)	n-fold
P04 - center	3.313	0.101	0.764	7.180	0.007	0.868	5.620	0.020	0.894	5.480	0.022	6.174
P04 - edge	3.702	0.077		7.384	0.006		5.781	0.018		2.854	0.138	
P07 - center	4.156	0.056	3.516	5.493	0.022	4.862	6.171	0.014	1.968	1.453	0.365	1.065
P07 - edge	2.342	0.197		3.211	0.108		5.195	0.027		1.362	0.389	
P11 - center	2.690	0.155	1.054	5.791	0.018	0.901	5.293	0.026	0.899	1.776	0.292	1.006
P11 - edge	2.614	0.163		5.942	0.016		5.446	0.023		1.768	0.294	
P15 - center	2.112	0.231	2.030	5.790	0.018	2.948	5.149	0.028	0.959	0.391	0.763	0.915
P15 - edge	1.090	0.470		4.231	0.053		5.210	0.027		0.519	0.698	
P16 - center	3.707	0.077	1.270	4.112	0.058	2.526	5.279	0.026	1.625	2.791	0.144	0.823
P16 - edge	3.362	0.097		2.775	0.146		4.579	0.042		3.073	0.119	
P19 - center	3.574	0.084	2.505	4.256	0.052	0.721	6.298	0.013	1.155	1.395	0.380	1.042
P19 - edge	2.249	0.210		4.728	0.038		6.090	0.015		1.335	0.396	
P23 - center	2.390	0.191	2.350	6.469	0.011	10.244	4.980	0.032	0.964	0.597	0.661	0.637
P23 - edge	1.158	0.448		3.112	0.116		5.034	0.031		1.248	0.421	
P24 - center	1.708	0.306	0.730	4.978	0.032	1.174	5.366	0.024	0.875	1.717	0.304	0.754
P24 - edge	2.162	0.223		4.746	0.037		5.558	0.021		2.125	0.229	
P26 - center	3.828	0.070	0.781	5.473	0.023	1.169	4.491	0.044	1.421	2.337	0.198	0.963
P26 - edge	4.184	0.055		5.248	0.026		3.985	0.063		2.391	0.191	
P27 - center	4.002	0.062	1.213	6.379	0.012	0.936	4.170	0.056	1.296	2.174	0.222	0.842
P27 - edge	3.723	0.076		6.474	0.011		3.797	0.072		2.422	0.187	
P30 - center	3.555	0.085	1.071	8.381	0.003	0.424	5.954	0.016	0.648	0.902	0.535	0.613
P30 - edge	3.456	0.091		9.618	0.001		6.579	0.010		1.607	0.328	
P36 - center	2.815	0.142	0.802	8.890	0.002	1.717	7.542	0.005	2.089	2.911	0.133	2.375
P36 - edge	3.133	0.114		8.110	0.004		6.480	0.011		1.663	0.316	
P21 - center	1.785	0.290	1.222	4.366	0.048	4.537	4.947	0.032	1.012	-0.840	1.790	0.823
P21 - edge	1.496	0.355		2.184	0.220		4.929	0.033		-0.559	1.474	
P34 - center	2.353	0.196	3.167	5.593	0.021	1.018	5.512	0.022	1.776	1.661	0.316	1.664
P34 - edge	0.690	0.620		5.567	0.021		4.683	0.039		0.926	0.526	
P35 - center	2.152	0.225	1.191	7.527	0.005	3.517	4.922	0.033	1.920	1.169	0.445	0.802
P35 - edge	1.900	0.268		5.712	0.019		3.981	0.063		1.487	0.357	

symbol of probe (P)	EphA5			H2BK			IGFBP5		
	ΔCt	2^-(ΔCt)	n-fold	ΔCt	2^-(ΔCt)	n-fold	ΔCt	2^-(ΔCt)	n-fold
P04 - center	6.342	0.012	17.914	1.816	0.284	0.169	-0.705	1.630	0.201
P04 - edge	2.179	0.221		4.379	0.048		1.607	0.328	
P07 - center	4.937	0.033	0.867	3.925	0.066	0.440	0.251	0.840	2.138
P07 - edge	5.144	0.028		5.109	0.029		-0.845	1.797	
P11 - center	4.441	0.046	0.896	4.429	0.046	0.873	-0.156	1.114	1.142
P11 - edge	4.599	0.041		4.625	0.041		-0.348	1.273	
P15 - center	4.147	0.056	1.048	3.830	0.070	0.938	-0.954	1.937	0.660
P15 - edge	4.080	0.059		3.923	0.066		-0.355	1.279	
P16 - center	1.706	0.306	0.611	4.356	0.049	2.104	0.743	0.597	1.419
P16 - edge	2.418	0.187		3.283	0.103		0.238	0.848	
P19 - center	7.138	0.007	1.259	4.157	0.056	1.553	-1.451	2.735	0.472
P19 - edge	6.806	0.009		3.521	0.087		-0.369	1.291	
P23 - center	4.518	0.044	0.609	3.115	0.115	1.245	-2.026	4.074	3.538
P23 - edge	5.235	0.027		2.799	0.144		-3.849	14.412	
P24 - center	4.721	0.038	0.541	3.356	0.098	0.563	0.238	0.848	4.600
P24 - edge	5.607	0.021		4.184	0.055		-1.963	3.900	
P26 - center	6.344	0.012	1.405	5.785	0.018	0.980	1.734	0.301	0.675
P26 - edge	5.853	0.017		5.813	0.018		2.301	0.203	
P27 - center	5.222	0.027	1.077	2.678	0.156	1.256	1.535	0.345	0.771
P27 - edge	5.114	0.029		2.350	0.196		1.910	0.266	
P30 - center	4.605	0.041	1.974	3.970	0.064	1.374	1.049	0.483	1.585
P30 - edge	3.623	0.081		3.512	0.088		0.384	0.766	
P36 - center	6.544	0.011	4.981	3.592	0.083	1.038	-1.004	2.005	0.236
P36 - edge	4.227	0.053		3.538	0.086		1.081	0.473	
P21 - center	3.742	0.075	1.312	4.698	0.039	0.897	-2.040	4.113	1.769
P21 - edge	3.350	0.098		4.855	0.035		-2.863	7.275	
P34 - center	4.803	0.036	1.583	4.228	0.053	1.552	-2.120	4.348	2.295
P34 - edge	4.140	0.057		3.595	0.083		-3.319	9.978	
P35 - center	7.090	0.007	1.601	3.549	0.085	0.326	-0.013	1.009	9.677
P35 - edge	6.411	0.012		5.168	0.028		-3.288	9.765	
