# Supplementary material for: Intratumoral Distribution of Lactate and the Monocarboxylate Transporters 1 and 4 in Human Glioblastoma Multiforme and Their Relationships to Tumor Progression-Associated Markers
Source: Int J Mol Sci. 2020 Aug 29;21(17):6254. doi: 10.3390/ijms21176254 (PMC7504270; doi:10.3390/ijms21176254)
Supplement: Supplementary file 1 [file ijms-21-06254-s001.zip › Table4S.pdf]

correlation analysis with clinical data

qPCR (15 patients enrolled)   yellow fields indicate statistically significant values

using the n-fold values:

	MCT1	MCT4	vimentin	GFAP	ß-catenin	KLF4	OCT4	Sox2	EPHA5	H2BK	IGFBP5
age	-0.146	0.233	0.342	0.568	0.240	0.452	0.397	-0.466	-0.474	0.426	0.417
gender (male: 1; female: 2)	0.012	-0.067	-0.237	-0.304	-0.354	-0.026	-0.670	0.183	0.255	-0.175	-0.037
symptoms before diagnosis	-0.151	-0.202	-0.294	0.139	0.078	0.422	-0.086	-0.136	-0.126	0.162	-0.056
primary versus recurrence (primary: 1; recurence: 2)	0.414	0.564	0.425	0.154	0.033	-0.157	0.173	-0.174	-0.141	0.024	0.332
time to relapse	0.263	0.536	0.373	0.030	-0.989	0.444	0.535	-0.959	0.041	-0.585	0.408
MGMT-status (unmethylated: 1; methylated: 2)	0.008	0.025	0.020	0.056	-0.133	0.135	-0.526	0.090	0.123	-0.333	0.345
1p19q-status (no codeletion:1; codeletion: 2)	-0.257	-0.296	-0.031	-0.233	-0.262	-0.228	0.034	-0.135	-0.171	0.471	-0.200

	OCT4 / gender	ß-catenin / time to relapse	Sox2 / time to relapse
p-value	-3.251	-24.311	-12.174
t-value	0.006	0.000	0.000

using the ΔC_T values:

		MCT1	MCT4	vimentin	GFAP	ß-catenin	KLF4	OCT4	Sox2	EPHA5	H2BK	IGFBP5
age	center + edge	-0.118	0.319	-0.087	-0.035	0.012	0.120	-0.074	-0.090	0.055	0.087	0.084
	center	0.008	0.441	0.075	-0.176	0.033	0.124	-0.269	-0.027	0.296	-0.432	0.193
	edge	-0.236	0.167	-0.221	0.030	0.004	0.153	0.047	-0.172	-0.227	0.786	0.068
gender (male: 1; female: 2)	center + edge	0.070	0.020	0.085	-0.212	-0.062	-0.259	-0.061	-0.231	-0.068	0.260	0.138
	center	0.068	-0.212	0.058	-0.250	-0.014	-0.393	0.189	-0.197	-0.178	0.548	-0.203
	edge	0.075	0.353	0.108	-0.227	-0.095	-0.294	-0.228	-0.278	0.058	-0.115	0.277
symptoms before diagnosis	center + edge	-0.217	-0.106	-0.015	0.081	0.008	0.010	0.196	-0.095	-0.241	-0.043	0.250
	center	-0.171	-0.081	0.163	-0.069	-0.139	-0.050	0.249	-0.060	-0.187	-0.140	0.212
	edge	-0.270	-0.148	-0.160	0.169	0.081	0.029	0.176	-0.142	-0.308	0.085	0.318
primary versus recurrence (primary: 1; recurrence: 2)	center + edge	-0.045	0.015	-0.160	-0.162	-0.136	-0.161	0.379	-0.122	-0.310	0.198	-0.016
	center	-0.196	-0.259	-0.340	-0.177	-0.170	0.031	0.314	-0.109	-0.256	0.110	-0.144
	edge	0.084	0.409	-0.015	-0.180	-0.140	-0.260	0.450	-0.141	-0.379	0.324	0.021
time to relapse	center + edge	0.913	-0.110	0.097	-0.742	0.070	-0.906	0.868	-0.442	0.553	0.318	0.189
	center	0.842	-0.899	-0.748	-0.960	0.266	-0.966	0.912	-0.363	0.618	0.811	-0.990
	edge	0.988	0.176	0.364	-0.912	-0.095	-0.911	0.963	-0.539	0.498	0.037	0.529
MGMT-status (unmethylated: 1; methylated: 2)	center + edge	0.341	-0.035	0.363	0.229	0.477	-0.103	0.216	0.308	-0.113	0.267	0.371
	center	0.385	-0.026	0.520	0.304	0.758	-0.315	0.335	0.369	-0.221	0.341	0.388
	edge	0.352	0.026	0.321	0.251	0.476	-0.068	0.122	0.286	0.028	0.127	0.479
1p19q-status (no codeletion:1; codeletion: 2)	center + edge	0.151	0.376	0.117	-0.341	-0.300	0.103	-0.053	-0.280	0.515	-0.205	-0.254
	center	0.298	0.658	0.226	-0.257	-0.246	0.316	-0.083	-0.280	0.684	-0.342	-0.330
	edge	0.041	0.182	0.038	-0.441	-0.373	0.080	-0.038	-0.285	0.324	-0.036	-0.297

	MCT1 / time to relapse			MCT4 / time to relapse	vimentin	GFAP		
	center + edge	center	edge	center	center	center + edge	center	edge
p-value	11.877	5.630	22.891	-7.385	-4.058	-5.848	-12.307	-8.004
t-value	0.000	0.000	0.000	0.000	0.001	0.000	0.000	0.000

	KLF4 / time to relapse			OCT4 / time to relapse			EPHA5 / time to relapse	H2BK / time to relapse
	center + edge	center	edge	center + edge	center	edge	center	center
p-value	-11.339	-13.412	-7.989	9.268	8.034	12.892	2.837	4.995
t-value	0.000	0.000	0.000	0.011	0.000	0.000	0.014	0.000

	relapse	MGMT	1p19q	EPHA5 / 1p19q
	center	center	center	center
p-value	-25.575	4.185	3.150	3.378
t-value	0.000	0.001	0.008	0.005
