# Supplementary material for: Intratumoral Distribution of Lactate and the Monocarboxylate Transporters 1 and 4 in Human Glioblastoma Multiforme and Their Relationships to Tumor Progression-Associated Markers
Source: Int J Mol Sci. 2020 Aug 29;21(17):6254. doi: 10.3390/ijms21176254 (PMC7504270; doi:10.3390/ijms21176254)
Supplement: Supplementary file 1 [file ijms-21-06254-s001.zip › Figure1S.pdf]

P11 - tumor
voxel 12;12

Row#12 Col#12 MR Hirn (2018.11.14 13:31:04) Subject 13 8.00e-01 mL, TE/TR/NS=288/1500/1 (s2DSI_TE288 TR1500)

Data of: Sektion Biomedizinische Bildgebung, Christian-Albrechts-Universitaet zu Kiel

LCModel (Version 6.3-1J) Copyright: S.W. Provencher. *Ref.: Magn. Reson. Med. 30:672-679 (1993).* *19-June-2019 17:44*

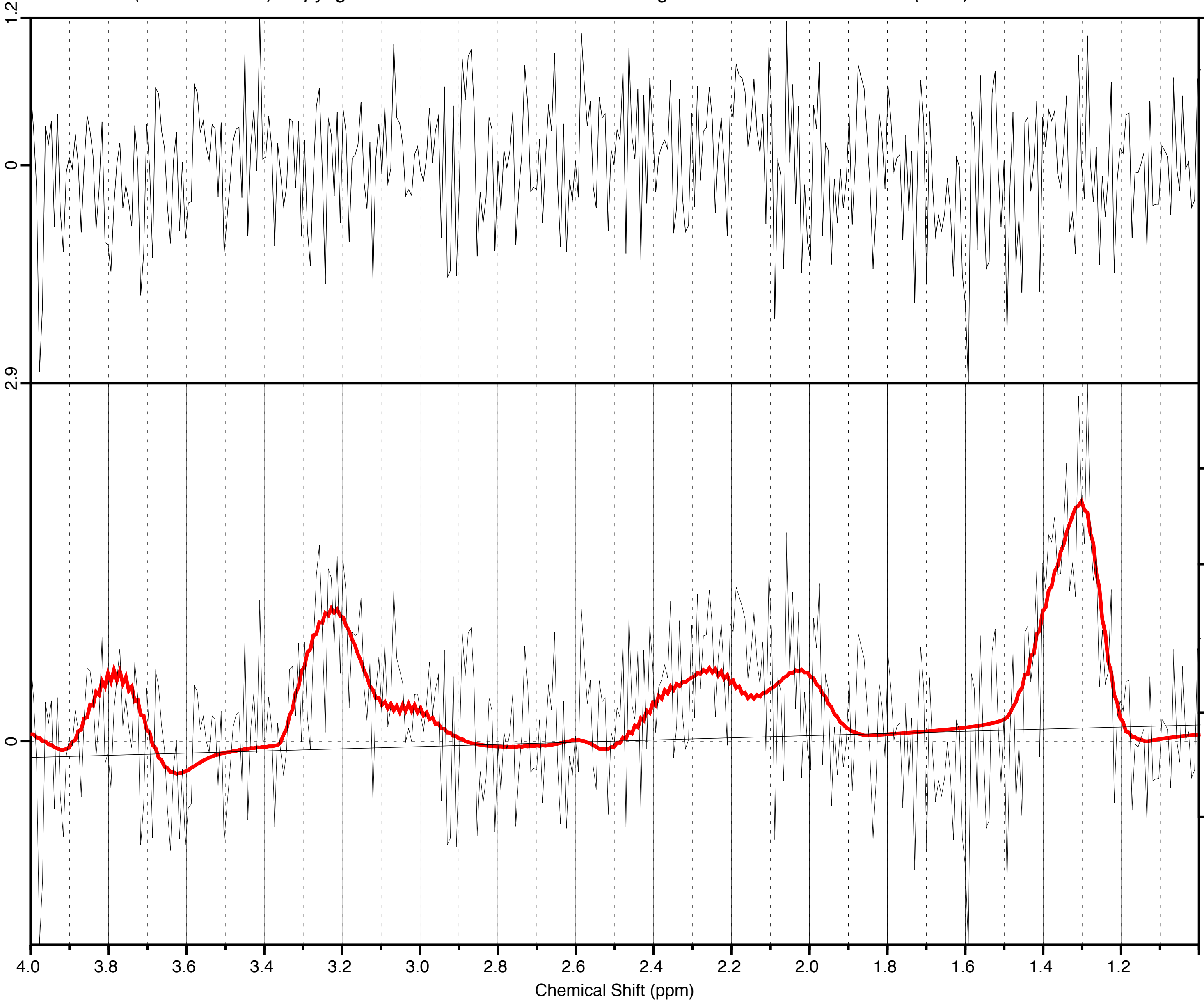

Conc.	%SD	/GPC+PC	Metabolite
0.000	999%	0.000	Ala
0.000	999%	0.000	Cr
22.984	56%	0.776	PCr
146.104	32%	4.933	Gln
0.000	999%	0.000	Glu
29.620	14%	1.000	GPC
0.000	999%	0.000	PCh
23.443	77%	0.791	GSH
0.000	999%	0.000	Ins
107.744	23%	3.638	Lac
36.733	29%	1.240	NAA
5.511	29%	0.186	NAAG
1.743	97%	5.9E-02	Scyllo
8.430	102%	0.285	Tau
9.511	103%	0.321	-CrCH2
29.620	14%	1.000	GPC+PCh
42.244	29%	1.426	NAA+NAAG
22.984	56%	0.776	Cr+PCr
146.104	32%	4.933	Glu+Gln
0.000	999%	0.000	Lip13a
53.262	52%	1.798	Lip13b
8.592	61%	0.290	Lip20
53.262	52%	1.798	Lip13a+Lip13b

DIAGNOSTICS

1 warning	STARTV 5
1 warning	STARTV 25
1 info	STARTV 24
7 info's	RFALSI 11
2 info's	RFALSI 4

MISCELLANEOUS OUTPUT

FWHM = 0.168 ppm S/N = 2
Data shift = 0.122 ppm
Ph: 18 deg -2.1 deg/ppm

INPUT CHANGES

```
deltat= 5.000e-04
echot= 288.00
filbas= '/home/stu111733/.lcmodel/
  basis-sets/gamma_press_te288_128
  mhz_662d.basis'
hzpppm= 1.2776e+02
icolen= 14
icolst= 10
```

P11 - tumor
voxel 12;12

Row#12 Col#12 MR Hirn (2018.11.14 13:31:04) Subject 13 8.00e-01 mL, TE/TR/NS=288/1500/1 (s2DSI_TE288 TR1500)

Data of: Sektion Biomedizinische Bildgebung, Christian-Albrechts-Universitaet zu Kiel

LCModel (Version 6.3-1J) Copyright: S.W. Provencher. *Ref.: Magn. Reson. Med. 30:672-679 (1993).* *19-June-2019 17:44*

Conc.	%SD	/GPC+PC	Metabolite
0.000	999%	0.000	Ala
0.000	999%	0.000	Cr
22.984	56%	0.776	PCr
146.104	32%	4.933	Gln
0.000	999%	0.000	Glu
29.620	14%	1.000	GPC
0.000	999%	0.000	PCh
23.443	77%	0.791	GSH
0.000	999%	0.000	Ins
107.744	23%	3.638	Lac
36.733	29%	1.240	NAA
5.511	29%	0.186	NAAG
1.743	97%	5.9E-02	Scyllo
8.430	102%	0.285	Tau
9.511	103%	0.321	-CrCH2
29.620	14%	1.000	GPC+PCh
42.244	29%	1.426	NAA+NAAG
22.984	56%	0.776	Cr+PCr
146.104	32%	4.933	Glu+Gln
0.000	999%	0.000	Lip13a
53.262	52%	1.798	Lip13b
8.592	61%	0.290	Lip20
53.262	52%	1.798	Lip13a+Lip13b

DIAGNOSTICS

1	warning	STARTV	5
1	warning	STARTV	25
1	info	STARTV	24
7	info's	RFALSI	11
2	info's	RFALSI	4

MISCELLANEOUS OUTPUT

FWHM = 0.168 ppm S/N = 2
Data shift = 0.122 ppm
Ph: 18 deg -2.1 deg/ppm

INPUT CHANGES

```
deltat= 5.000e-04
echot= 288.00
filbas= '/home/stu111733/.lcmodel/basis-sets/gam
  ma_press_te288_128mhz_662d.basis'
hzpppm= 1.2776e+02
icolen= 14
icolst= 10
irowen= 13
irowst= 9
islice= 1
lps= 8
ltable= 7
ndcols= 16
ndrows= 20
ndslic= 1
nunfil= 1024
ppmend= 1.0
ppmst= 4.0
savdir= '/home/data/Studies/GBM_Spektro/
results/
   s13/'
sptype= 'tumor'
srcraw= '/home/data/Studies/GBM_Spektro/data/ [illegible]
s13
```

P11 - edema
voxel 10;11

Row#10 Col#11 MR Hirn (2018.11.14 13:31:04) Subject 13 8.00e-01 mL, TE/TR/NS=288/1500/1 (s2DSI_TE288 TR1500)

Data of: Sektion Biomedizinische Bildgebung, Christian-Albrechts-Universitaet zu Kiel

LCModel (Version 6.3-1J) Copyright: S.W. Provencher. Ref.: Magn. Reson. Med. 30:672-679 (1993). *19-June-2019 17:44*

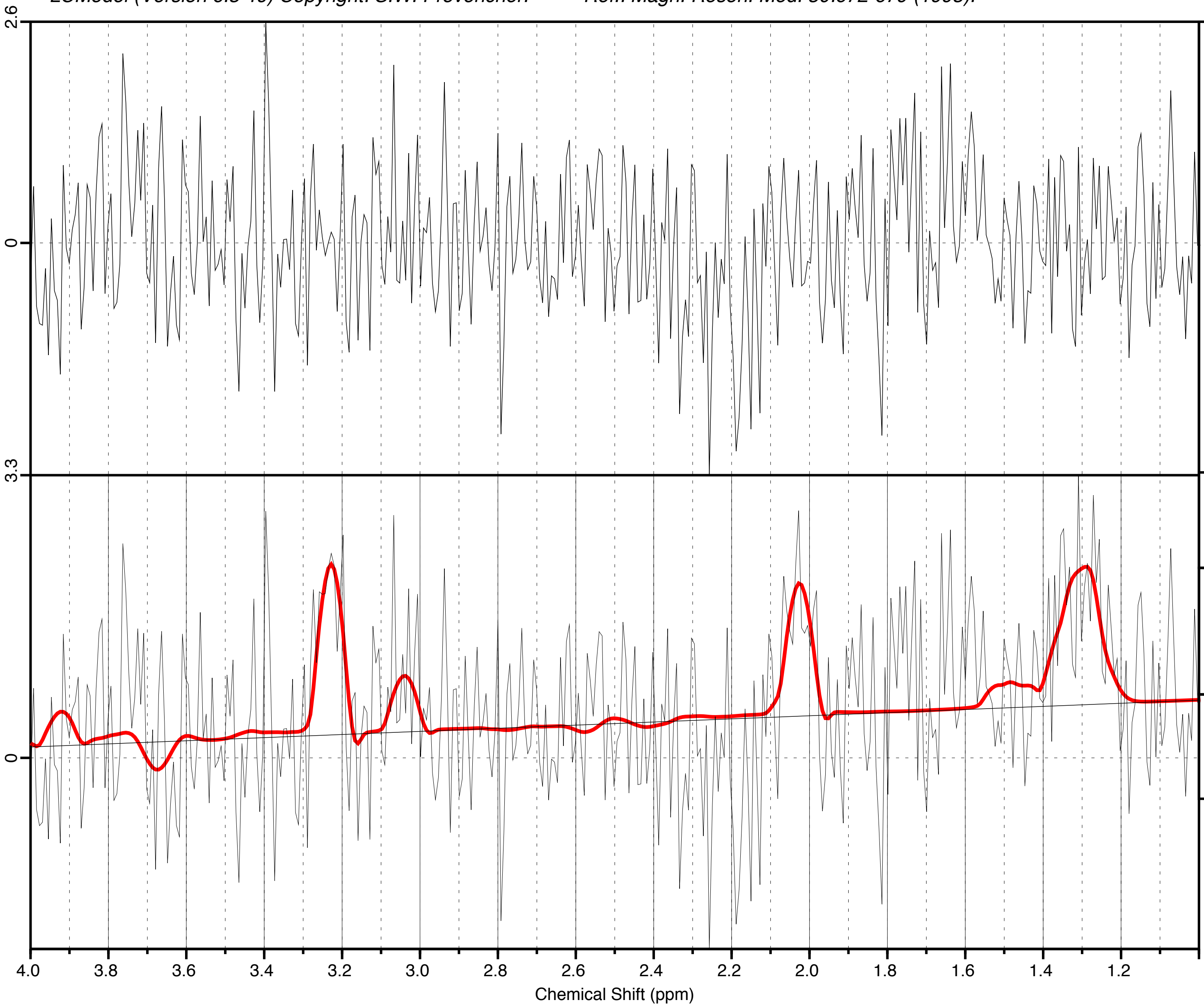

Conc.	%SD	/GPC+PC	Metabolite
18.849	100%	0.860	Ala
21.895	53%	0.999	Cr
0.000	999%	0.000	PCr
0.000	999%	0.000	Gln
4.517	789%	0.206	Glu
21.918	20%	1.000	GPC
0.000	999%	0.000	PCh
0.000	999%	0.000	GSH
0.000	999%	0.000	Ins
51.948	48%	2.370	Lac
47.568	26%	2.170	NAA
6.829	75%	0.312	NAAG
1.330	92%	6.1E-02	Scyllo
7.822	77%	0.357	Tau
4.199	108%	0.192	-CrCH2
21.918	20%	1.000	GPC+PCh
54.397	25%	2.482	NAA+NAAG
21.895	53%	0.999	Cr+PCr
4.517	789%	0.206	Glu+Gln
0.000	999%	0.000	Lip13a
68.031	55%	3.104	Lip13b
8.498	94%	0.388	Lip20
68.031	55%	3.104	Lip13a+Lip13b

DIAGNOSTICS

5	info's	RFALSI	11
5	info's	RFALSI	4
3	info's	TWOREG	8
1	info	TWOREG	9

MISCELLANEOUS OUTPUT

FWHM = 0.069 ppm S/N = 1
Data shift = 0.084 ppm
Ph: 43 deg -3.9 deg/ppm

INPUT CHANGES

deltat= 5.000e-04
echot= 288.00
filbas= '/home/stu111733/.lcmodel/basis-sets/gamma_press_te288_128mhz_662d.basis'
hzpppm= 1.2776e+02
icolen= 14
icolst= 10
irowen= 13

P11 - edema
voxel 10;11

Row#10 Col#11 MR Hirn (2018.11.14 13:31:04) Subject 13 8.00e-01 mL, TE/TR/NS=288/1500/1 (s2DSI_TE288 TR1500)

Data of: Sektion Biomedizinische Bildgebung, Christian-Albrechts-Universitaet zu Kiel

LCModel (Version 6.3-1J) Copyright: S.W. Provencher.   *Ref.: Magn. Reson. Med. 30:672-679 (1993).*   *19-June-2019 17:44*

Conc.	%SD	/GPC+PC	Metabolite
18.849	100%	0.860	Ala
21.895	53%	0.999	Cr
0.000	999%	0.000	PCr
0.000	999%	0.000	Gln
4.517	789%	0.206	Glu
21.918	20%	1.000	GPC
0.000	999%	0.000	PCh
0.000	999%	0.000	GSH
0.000	999%	0.000	Ins
51.948	48%	2.370	Lac
47.568	26%	2.170	NAA
6.829	75%	0.312	NAAG
1.330	92%	6.1E-02	Scyllo
7.822	77%	0.357	Tau
4.199	108%	0.192	-CrCH2
21.918	20%	1.000	GPC+PCh
54.397	25%	2.482	NAA+NAAG
21.895	53%	0.999	Cr+PCr
4.517	789%	0.206	Glu+Gln
0.000	999%	0.000	Lip13a
68.031	55%	3.104	Lip13b
8.498	94%	0.388	Lip20
68.031	55%	3.104	Lip13a+Lip13b

DIAGNOSTICS

```
5 info's    RFALSI 11
5 info's    RFALSI  4
3 info's    TWOREG  8
1 info      TWOREG  9
```

MISCELLANEOUS OUTPUT

```
FWHM = 0.069 ppm    S/N =   1
Data shift = 0.084 ppm
Ph:  43 deg      -3.9 deg/ppm
```

INPUT CHANGES

```
deltat= 5.000e-04
echot= 288.00
filbas= '/home/stu111733/.lcmodel/basis-sets/gam
  ma_press_te288_128mhz_662d.basis'
hzpppm= 1.2776e+02
icolen= 14
icolst= 10
irowen= 13
irowst= 9
islice= 1
lps= 8
ltable= 7
ndcols= 16
ndrows= 20
ndslic= 1
nunfil= 1024
ppmend= 1.0
ppmst= 4.0
savdir= '/home/data/Studies/GBM_Spektro/
results/
   s13/'
sptype= 'tumor'
sr[illegible]_3d1_raw_act.SDAT'
```

P12 - tumor voxel 19;8

Row#19 Col#8 MR Spektroskopie (2018.11.20 09:44:02) Subject 01 8.00e-01 mL, TE/TR/NS=288/1500/1 (s2DSI_TE288 TR1500)

Data of: Sektion Biomedizinische Bildgebung, Christian-Albrechts-Universitaet zu Kiel

LCModel (Version 6.3-1J) Copyright: S.W. Provencher.   *Ref.: Magn. Reson. Med. 30:672-679 (1993).*   *13-June-2019 09:33*

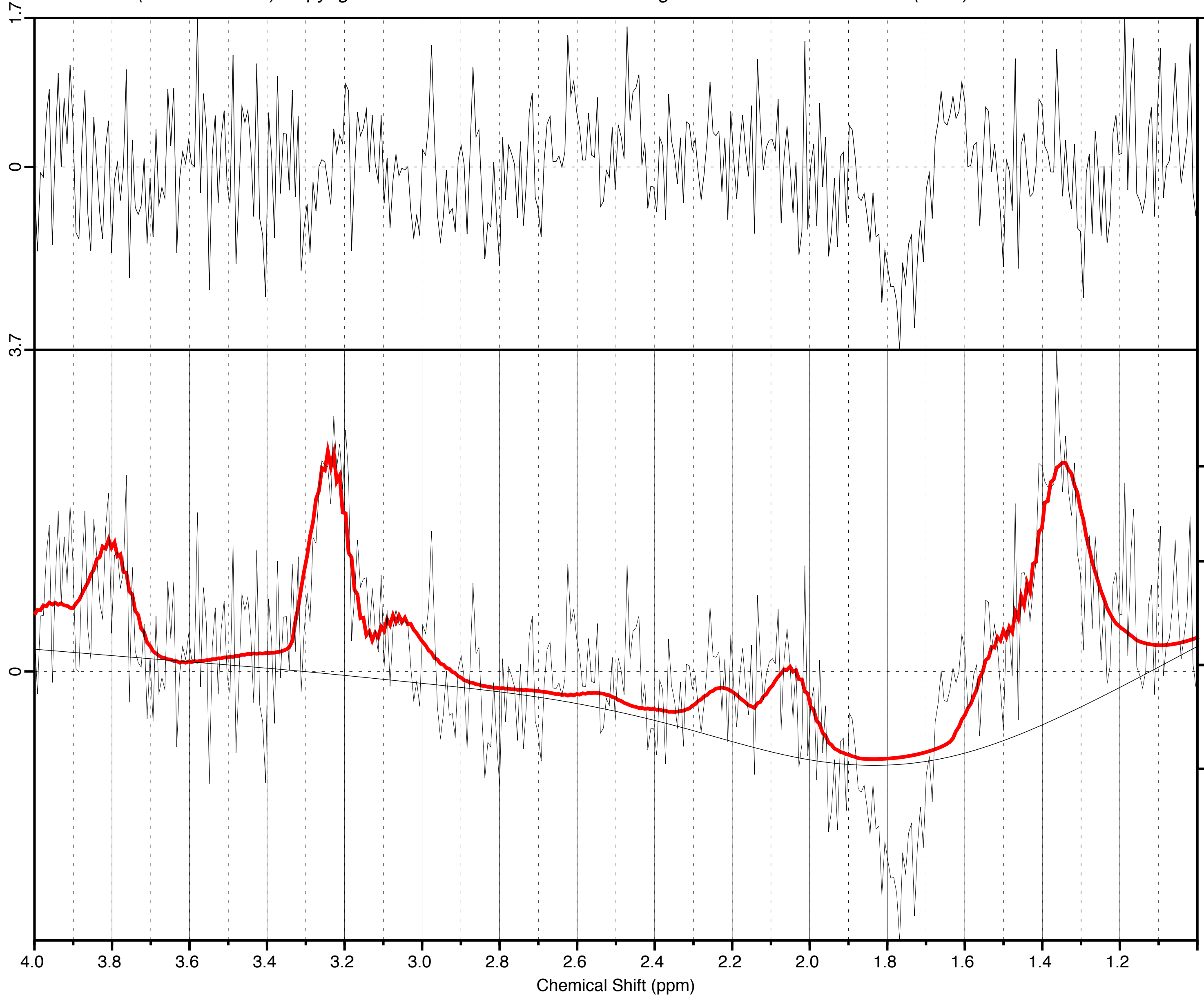

Conc.	%SD	/GPC+PC	Metabolite
78.510	29%	1.609	Ala
40.990	34%	0.840	Cr
0.000	999%	0.000	PCr
16.483	536%	0.338	Gln
1.685	999%	3.5E-02	Glu
0.000	999%	0.000	GPC
**48.805**	**12%**	**1.000**	**PCh**
60.273	38%	1.235	GSH
0.000	999%	0.000	Ins
152.575	18%	3.126	Lac
44.705	33%	0.916	NAA
6.707	33%	0.137	NAAG
2.195	98%	4.5E-02	Scyllo
9.312	117%	0.191	Tau
2.433	307%	5.0E-02	-CrCH2
**48.805**	**12%**	**1.000**	**GPC+PCh**
51.412	33%	1.053	NAA+NAAG
40.990	34%	0.840	Cr+PCr
18.168	285%	0.372	Glu+Gln
223.030	54%	4.570	Lip13a
0.000	999%	0.000	Lip13b
41.348	100%	0.847	Lip20
223.030	54%	4.570	Lip13a+Lip13b

DIAGNOSTICS

```
1 info      STARTV 24
2 info's    TWOREG  8
3 info's    RFALSI 11
```

MISCELLANEOUS OUTPUT

```
FWHM = 0.107 ppm    S/N =   2
Data shift = 0.061 ppm
Ph:  19 deg        1.4 deg/ppm
```

INPUT CHANGES

```
deltat= 5.000e-04
echot= 288.00
filbas= '/home/stu111733/.lcmodel/
  basis-sets/gamma_press_te288_128
  mhz_662d.basis'
hzpppm= 1.2776e+02
icolen= 9
icolst= 7
irowen= 20
irowst= 17
```

P12 - tumor
voxel 19;8

Row#19 Col#8 MR Spektroskopie (2018.11.20 09:44:02) Subject 01 8.00e-01 mL, TE/TR/NS=288/1500/1 (s2DSI_TE288 TR1500)

Data of: Sektion Biomedizinische Bildgebung, Christian-Albrechts-Universitaet zu Kiel

LCModel (Version 6.3-1J) Copyright: S.W. Provencher. *Ref.: Magn. Reson. Med. 30:672-679 (1993).* *13-June-2019 09:33*

Conc.	%SD	/GPC+PC	Metabolite
78.510	29%	1.609	Ala
40.990	34%	0.840	Cr
0.000	999%	0.000	PCr
16.483	536%	0.338	Gln
1.685	999%	3.5E-02	Glu
0.000	999%	0.000	GPC
**48.805**	**12%**	**1.000**	**PCh**
60.273	38%	1.235	GSH
0.000	999%	0.000	Ins
152.575	18%	3.126	Lac
44.705	33%	0.916	NAA
6.707	33%	0.137	NAAG
2.195	98%	4.5E-02	Scyllo
9.312	117%	0.191	Tau
2.433	307%	5.0E-02	-CrCH2
**48.805**	**12%**	**1.000**	**GPC+PCh**
51.412	33%	1.053	NAA+NAAG
40.990	34%	0.840	Cr+PCr
18.168	285%	0.372	Glu+Gln
223.030	54%	4.570	Lip13a
0.000	999%	0.000	Lip13b
41.348	100%	0.847	Lip20
223.030	54%	4.570	Lip13a+Lip13b

DIAGNOSTICS

```
1 info      STARTV 24
2 info's    TWOREG  8
3 info's    RFALSI 11
```

MISCELLANEOUS OUTPUT

```
FWHM = 0.107 ppm    S/N =   2
Data shift = 0.061 ppm
Ph:  19 deg        1.4 deg/ppm
```

INPUT CHANGES

```
deltat= 5.000e-04
echot= 288.00
filbas= '/home/stu111733/.lcmodel/basis-sets/gam
  ma_press_te288_128mhz_662d.basis'
hzpppm= 1.2776e+02
icolen= 9
icolst= 7
irowen= 20
irowst= 17
islice= 1
lps= 8
ltable= 7
ndcols= 16
ndrows= 20
ndslic= 1
nunfil= 1024
ppmend= 1.0
ppmst= 4.0
savdir= '/home/data/Studies/GBM_Spektro/
results/s01/'
sptype= 'tumor'
srcraw= '/home/data/Studies/GBM_Spektro/data/
s01/DIEX_5_1_raw_act.SDAT'
```

P12 - edema
voxel 19;10

Row#19 Col#10 MR Spektroskopie (2018.11.20 09:44:02) Subject 01 8.00e-01 mL, TE/TR/NS=288/1500/1 (s2DSI_TE288 TR1500)

Data of: Sektion Biomedizinische Bildgebung, Christian-Albrechts-Universitaet zu Kiel

LCModel (Version 6.3-1J) Copyright: S.W. Provencher.   Ref.: Magn. Reson. Med. 30:672-679 (1993).   *26-November-2019 17:23*

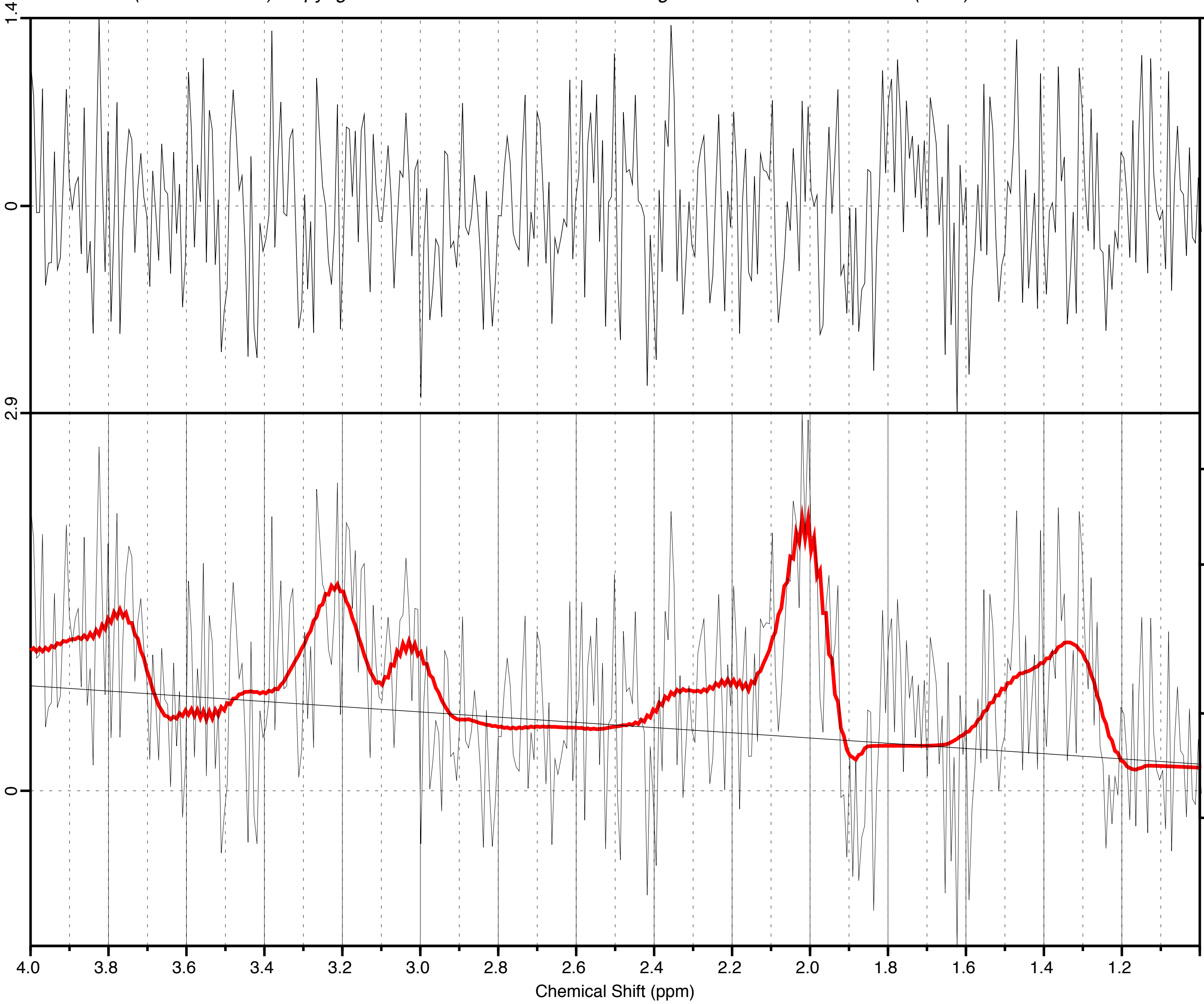

Conc.	%SD	/GPC+PC	Metabolite
41.515	39%	2.082	Ala
16.603	459%	0.833	Cr
17.408	434%	0.873	PCr
86.308	57%	4.328	Gln
0.000	999%	0.000	Glu
11.697	183%	0.587	GPC
8.244	264%	0.413	PCh
17.146	107%	0.860	GSH
98.807	152%	4.955	Ins
65.962	22%	3.308	Lac
**95.151**	**14%**	**4.772**	**NAA**
13.532	47%	0.679	NAAG
1.972	56%	9.9E-02	Scyllo
9.227	60%	0.463	Tau
2.377	270%	0.119	-CrCH2
19.940	22%	1.000	GPC+PCh
**108.683**	**13%**	**5.450**	**NAA+NAAG**
34.011	38%	1.706	Cr+PCr
86.308	57%	4.328	Glu+Gln
0.000	999%	0.000	Lip13a
0.000	999%	0.000	Lip13b
0.000	999%	0.000	Lip20
0.000	999%	0.000	Lip13a+Lip13b

DIAGNOSTICS

**1 warning**	**STARTV 25**
1 info	STARTV 24
7 info's	RFALSI 11
4 info's	RFALSI 4
2 info's	TWOREG 8

MISCELLANEOUS OUTPUT

FWHM = 0.122 ppm   S/N =   1
Data shift = 0.008 ppm
Ph: -1 deg   4.0 deg/ppm

INPUT CHANGES

deltat= 5.000e-04
echot= 288.00
filbas= '/home/stu111733/.lcmodel/basis-sets/gamma_press_te288_128mhz_662d.basis'
hzpppm= 1.2776e+02
icolen= 11
icolst= 10

P12 - edema
voxel 19;10

Row#19 Col#10 MR Spektroskopie (2018.11.20 09:44:02) Subject 01 8.00e-01 mL, TE/TR/NS=288/1500/1 (s2DSI_TE288 TR1500)

Data of: Sektion Biomedizinische Bildgebung, Christian-Albrechts-Universitaet zu Kiel

LCModel (Version 6.3-1J) Copyright: S.W. Provencher. *Ref.: Magn. Reson. Med. 30:672-679 (1993).* *26-November-2019 17:23*

Conc.	%SD	/GPC+PC	Metabolite
41.515	39%	2.082	Ala
16.603	459%	0.833	Cr
17.408	434%	0.873	PCr
86.308	57%	4.328	Gln
0.000	999%	0.000	Glu
11.697	183%	0.587	GPC
8.244	264%	0.413	PCh
17.146	107%	0.860	GSH
98.807	152%	4.955	Ins
65.962	22%	3.308	Lac
**95.151**	**14%**	**4.772**	**NAA**
13.532	47%	0.679	NAAG
1.972	56%	9.9E-02	Scyllo
9.227	60%	0.463	Tau
2.377	270%	0.119	-CrCH2
19.940	22%	1.000	GPC+PCh
**108.683**	**13%**	**5.450**	**NAA+NAAG**
34.011	38%	1.706	Cr+PCr
86.308	57%	4.328	Glu+Gln
0.000	999%	0.000	Lip13a
0.000	999%	0.000	Lip13b
0.000	999%	0.000	Lip20
0.000	999%	0.000	Lip13a+Lip13b

DIAGNOSTICS

**1 warning**	**STARTV 25**
1 info	STARTV 24
7 info's	RFALSI 11
4 info's	RFALSI 4
2 info's	TWOREG 8

MISCELLANEOUS OUTPUT

FWHM = 0.122 ppm S/N = 1
Data shift = 0.008 ppm
Ph: -1 deg 4.0 deg/ppm

INPUT CHANGES

deltat= 5.000e-04
echot= 288.00
filbas= '/home/stu111733/.lcmodel/basis-sets/gamma_press_te288_128mhz_662d.basis'
hzpppm= 1.2776e+02
icolen= 11

icolst= 10
irowen= 20
irowst= 17
islice= 1
lps= 8
ltable= 7
ndcols= 16
ndrows= 20
ndslic= 1
nunfil= 1024
ppmend= 1.0
ppmst= 4.0
savdir= '/home/stu111733/.lcmodel/saved/MR_Spektroskopie/s01/2018.11.20_09:44:02/' sptype= 'tumor'
srcraw= '/home/data/Studies/GBM_Spektro/data/s01/DBIEX_5_1_raw_act.SDAT'

P15 - tumor
voxel 10;4

Row#10 Col#4 MR Hirn (2019.01.04 14:00:10) Subject 06 6.30e-01 mL, TE/TR/NS=288/1500/4 (s2DSI_TE288 TR1500)

Data of: Sektion Biomedizinische Bildgebung, Christian-Albrechts-Universitaet zu Kiel

LCModel (Version 6.3-1J) Copyright: S.W. Provencher. Ref.: Magn. Reson. Med. 30:672-679 (1993). *13-June-2019 10:47*

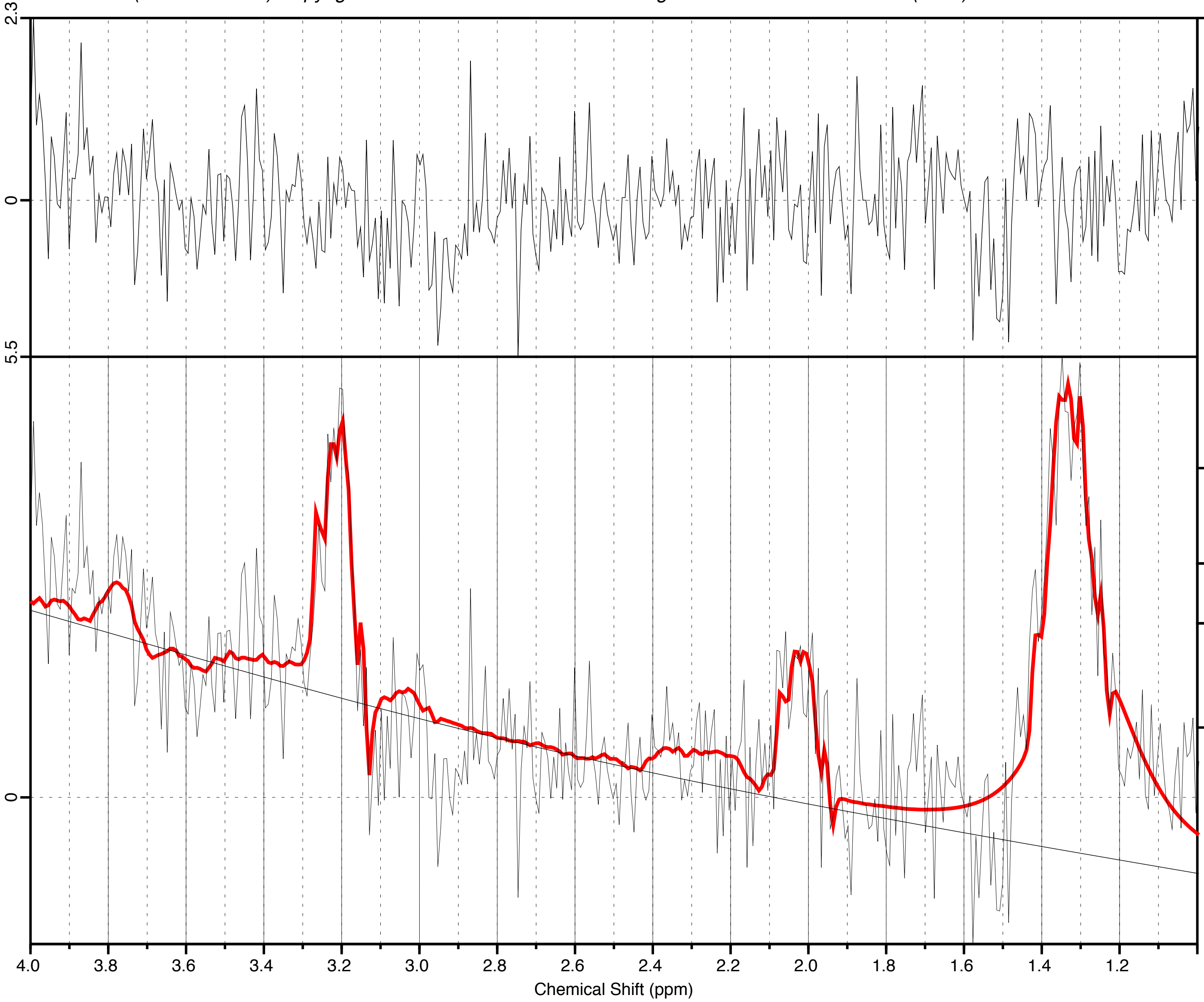

Conc.	%SD	/GPC+PC	Metabolite
0.000	999%	0.000	Ala
11.503	311%	0.243	Cr
1.725	999%	3.6E-02	PCr
49.175	98%	1.038	Gln
27.302	156%	0.576	Glu
0.000	999%	0.000	GPC
47.396	10%	1.000	PCh
12.177	123%	0.257	GSH
58.279	163%	1.230	Ins
200.884	13%	4.238	Lac
67.630	19%	1.427	NAA
13.176	59%	0.278	NAAG
3.028	81%	6.4E-02	Scyllo
16.636	73%	0.351	Tau
0.000	999%	0.000	-CrCH2
47.396	10%	1.000	GPC+PCh
80.806	17%	1.705	NAA+NAAG
13.228	81%	0.279	Cr+PCr
76.477	52%	1.614	Glu+Gln
723.821	20%	15.272	Lip13a
0.000	999%	0.000	Lip13b
22.525	231%	0.475	Lip20
723.821	20%	15.272	Lip13a+Lip13b

DIAGNOSTICS

1 info FINOUT 9

MISCELLANEOUS OUTPUT

FWHM = 0.092 ppm S/N = 4
Data shift = 0.015 ppm
Ph: -29 deg -11.1 deg/ppm

INPUT CHANGES

```
deltat= 5.000e-04
echot= 288.00
filbas= '/home/stu111733/.lcmodel/
  basis-sets/gamma_press_te288_128
  mhz_662d.basis'
hzpppm= 1.2776e+02
icolen= 7
icolst= 2
irowen= 11
irowst= 7
islice= 1
lps= 8
```

P15 - tumor
voxel 10;4

Row#10 Col#4 MR Hirn (2019.01.04 14:00:10) Subject 06 6.30e-01 mL, TE/TR/NS=288/1500/4 (s2DSI_TE288 TR1500)

Data of: Sektion Biomedizinische Bildgebung, Christian-Albrechts-Universitaet zu Kiel

LCModel (Version 6.3-1J) Copyright: S.W. Provencher. *Ref.: Magn. Reson. Med. 30:672-679 (1993).* *13-June-2019 10:47*

Conc.	%SD	/GPC+PC	Metabolite
0.000	999%	0.000	Ala
11.503	311%	0.243	Cr
1.725	999%	3.6E-02	PCr
49.175	98%	1.038	Gln
27.302	156%	0.576	Glu
0.000	999%	0.000	GPC
47.396	10%	1.000	PCh
12.177	123%	0.257	GSH
58.279	163%	1.230	Ins
200.884	13%	4.238	Lac
67.630	19%	1.427	NAA
13.176	59%	0.278	NAAG
3.028	81%	6.4E-02	Scyllo
16.636	73%	0.351	Tau
0.000	999%	0.000	-CrCH2
47.396	10%	1.000	GPC+PCh
80.806	17%	1.705	NAA+NAAG
13.228	81%	0.279	Cr+PCr
76.477	52%	1.614	Glu+Gln
723.821	20%	15.272	Lip13a
0.000	999%	0.000	Lip13b
22.525	231%	0.475	Lip20
723.821	20%	15.272	Lip13a+Lip13b

DIAGNOSTICS

1 info FINOUT 9

MISCELLANEOUS OUTPUT

FWHM = 0.092 ppm S/N = 4
Data shift = 0.015 ppm
Ph: -29 deg -11.1 deg/ppm

INPUT CHANGES

deltat= 5.000e-04
echot= 288.00
filbas= '/home/stu111733/.lcmodel/basis-sets/gamma_press_te288_128mhz_662d.basis'
hzpppm= 1.2776e+02
icolen= 7
icolst= 2
irowen= 11
irowst= 7
islice= 1

lps= 8
ltable= 7
ndcols= 12
ndrows= 14
ndslic= 1
nunfil= 1024
ppmend= 1.0
ppmst= 4.0
savdir= '/home/data/Studies/GBM_Spektro/results/'
sptype= 'tumor'
srcraw= '/home/data/Studies/GBM_Spektro/data/$DBIEX_7_1_raw_act.SDAT'

P15 - edema
voxel 11;3

Row#11 Col#3 MR Hirn (2019.01.04 14:00:10) Subject 06 6.30e-01 mL, TE/TR/NS=288/1500/4 (s2DSI_TE288 TR1500)

Data of: Sektion Biomedizinische Bildgebung, Christian-Albrechts-Universitaet zu Kiel

LCModel (Version 6.3-1J) Copyright: S.W. Provencher. Ref.: Magn. Reson. Med. 30:672-679 (1993). *13-June-2019 10:48*

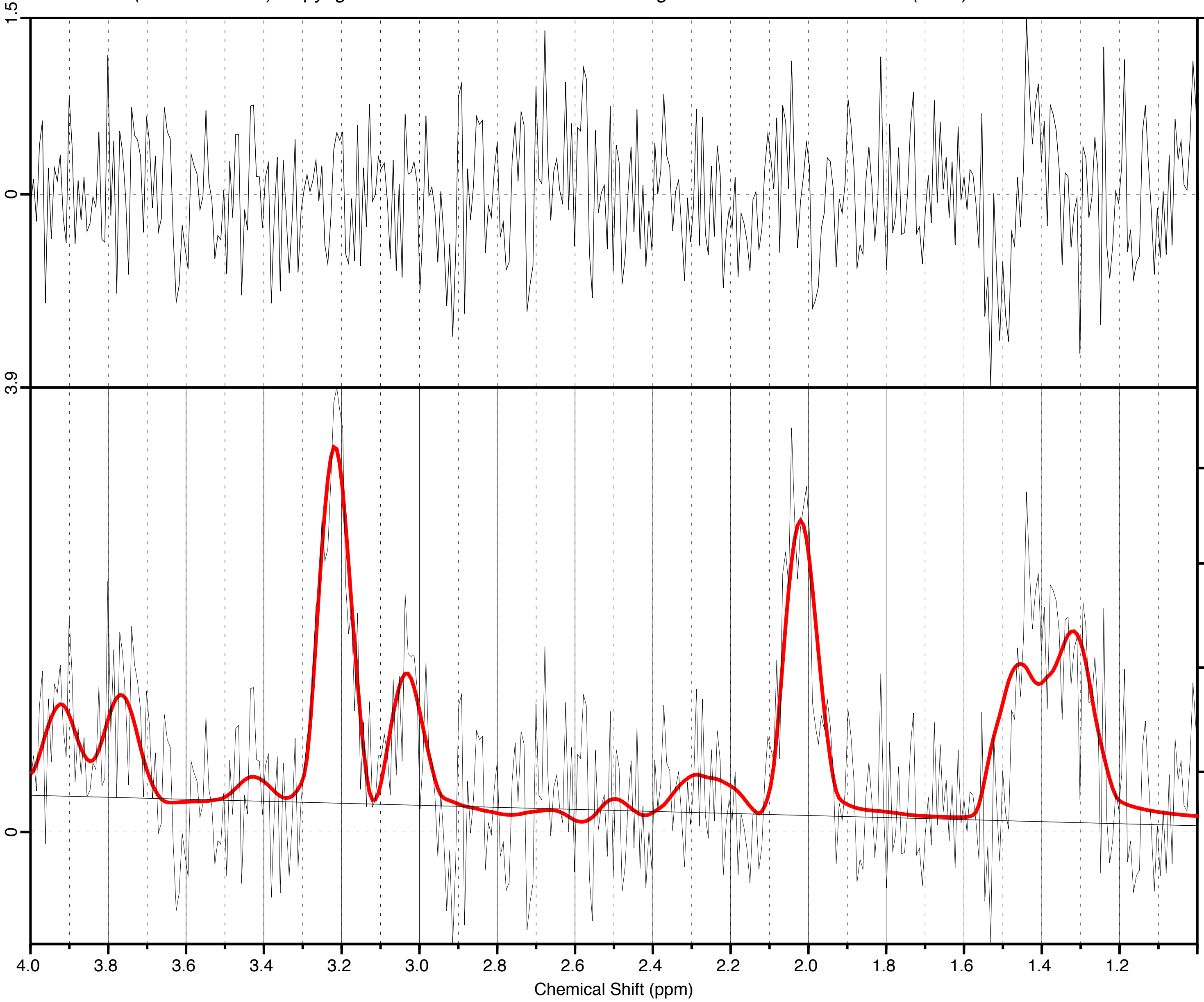

Conc.	%SD	/GPC+PC	Metabolite
97.510	15%	2.098	Ala
43.827	108%	0.943	Cr
10.286	462%	0.221	PCr
16.852	280%	0.363	Gln
52.430	76%	1.128	Glu
0.000	999%	0.000	GPC
**46.481**	**8%**	**1.000**	**PCh**
15.546	89%	0.334	GSH
0.000	999%	0.000	Ins
88.174	23%	1.897	Lac
**96.973**	**13%**	**2.086**	**NAA**
31.006	32%	0.667	NAAG
0.657	448%	1.4E-02	Scyllo
25.707	58%	0.553	Tau
2.439	462%	5.2E-02	-CrCH2
**46.481**	**8%**	**1.000**	**GPC+PCh**
**127.979**	**8%**	**2.753**	**NAA+NAAG**
54.113	18%	1.164	Cr+PCr
69.282	51%	1.491	Glu+Gln
12.125	712%	0.261	Lip13a
0.000	999%	0.000	Lip13b
0.246	999%	5.3E-03	Lip20
12.125	712%	0.261	Lip13a+Lip13b

DIAGNOSTICS

9 info's	RFALSI	11
4 info's	RFALSI	4
2 info's	TWOREG	8

MISCELLANEOUS OUTPUT

FWHM = 0.092 ppm S/N = 3
Data shift = 0.046 ppm
Ph: 22 deg -2.3 deg/ppm

INPUT CHANGES

deltat= 5.000e-04
echot= 288.00
filbas= '/home/stu111733/.lcmodel/basis-sets/gamma_press_te288_128mhz_662d.basis'
hzpppm= 1.2776e+02
icolen= 7
icolst= 2
irowen= 11
irowst= 7

P15 - edema
voxel 11;3

Row#11 Col#3 MR Hirn (2019.01.04 14:00:10) Subject 06 6.30e-01 mL, TE/TR/NS=288/1500/4 (s2DSI_TE288 TR1500)

Data of: Sektion Biomedizinische Bildgebung, Christian-Albrechts-Universitaet zu Kiel

LCModel (Version 6.3-1J) Copyright: S.W. Provencher. *Ref.: Magn. Reson. Med. 30:672-679 (1993).* *13-June-2019 10:48*

Conc.	%SD	/GPC+PC	Metabolite
97.510	15%	2.098	Ala
43.827	108%	0.943	Cr
10.286	462%	0.221	PCr
16.852	280%	0.363	Gln
52.430	76%	1.128	Glu
0.000	999%	0.000	GPC
**46.481**	**8%**	**1.000**	**PCh**
15.546	89%	0.334	GSH
0.000	999%	0.000	Ins
88.174	23%	1.897	Lac
**96.973**	**13%**	**2.086**	**NAA**
31.006	32%	0.667	NAAG
0.657	448%	1.4E-02	Scyllo
25.707	58%	0.553	Tau
2.439	462%	5.2E-02	-CrCH2
**46.481**	**8%**	**1.000**	**GPC+PCh**
**127.979**	**8%**	**2.753**	**NAA+NAAG**
54.113	18%	1.164	Cr+PCr
69.282	51%	1.491	Glu+Gln
12.125	712%	0.261	Lip13a
0.000	999%	0.000	Lip13b
0.246	999%	5.3E-03	Lip20
12.125	712%	0.261	Lip13a+Lip13b

DIAGNOSTICS

```
9 info's     RFALSI 11
4 info's     RFALSI  4
2 info's     TWOREG  8
```

MISCELLANEOUS OUTPUT

```
FWHM = 0.092 ppm    S/N =   3
Data shift = 0.046 ppm
Ph:  22 deg       -2.3 deg/ppm
```

INPUT CHANGES

```
deltat= 5.000e-04
echot= 288.00
filbas= '/home/stu111733/.lcmodel/basis-sets/gam
  ma_press_te288_128mhz_662d.basis'
hzpppm= 1.2776e+02
icolen= 7
icolst= 2
irowen= 11
irowst= 7
islice= 1
lps= 8
ltable= 7
ndcols= 12
ndrows= 14
ndslic= 1
nunfil= 1024
ppmend= 1.0
ppmst= 4.0
savdir= '/home/data/Studies/GBM_Spektro/results/
  '
sptype= 'tumor'
srcraw= '/home/data/Studies/GBM_Spektro/data/
$DBIEX_7_1_raw_act.SDAT'
```

P19 - tumor
voxel 12;7

Row#12 Col#7 MR Hirn (2019.01.09 17:36:15) Subject 10 7.86e-01 mL, TE/TR/NS=288/1500/3 (s2DSI_TE288 TR1500)

Data of: Sektion Biomedizinische Bildgebung, Christian-Albrechts-Universitaet zu Kiel

LCModel (Version 6.3-1J) Copyright: S.W. Provencher. Ref.: Magn. Reson. Med. 30:672-679 (1993). *14-June-2019 17:23*

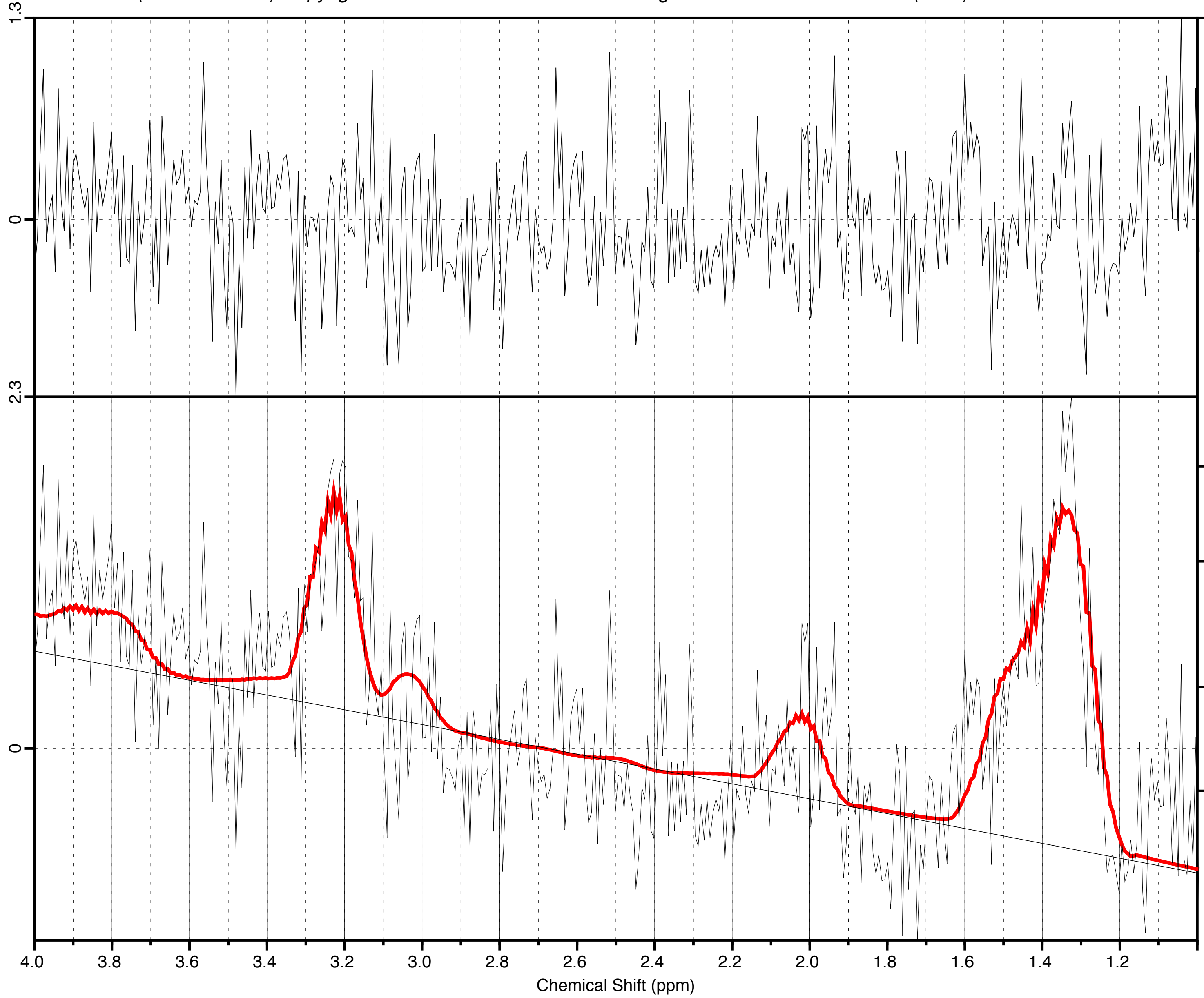

Conc.	%SD	/GPC+PC	Metabolite
**88.337**	**14%**	**3.139**	**Ala**
20.430	42%	0.726	Cr
0.000	999%	0.000	PCr
0.000	999%	0.000	Gln
0.000	999%	0.000	Glu
0.000	999%	0.000	GPC
**28.142**	**11%**	**1.000**	**PCh**
5.852	160%	0.208	GSH
0.000	999%	0.000	Ins
**152.662**	**8%**	**5.425**	**Lac**
29.949	29%	1.064	NAA
3.323	127%	0.118	NAAG
1.650	74%	5.9E-02	Scyllo
5.733	107%	0.204	Tau
0.000	999%	0.000	-CrCH2
**28.142**	**11%**	**1.000**	**GPC+PCh**
33.272	27%	1.182	NAA+NAAG
20.430	42%	0.726	Cr+PCr
0.000	999%	0.000	Glu+Gln
0.000	999%	0.000	Lip13a
0.000	999%	0.000	Lip13b
0.000	999%	0.000	Lip20
0.000	999%	0.000	Lip13a+Lip13b

DIAGNOSTICS

```
1 info      STARTV 24
6 info's    RFALSI 11
4 info's    RFALSI  4
2 info's    TWOREG  8
```

MISCELLANEOUS OUTPUT

```
FWHM = 0.138 ppm    S/N =   2
Data shift = 0.054 ppm
Ph: -17 deg     -16.1 deg/ppm
```

INPUT CHANGES

```
deltat= 5.000e-04
echot= 288.00
filbas= '/home/stu111733/.lcmodel/
  basis-sets/gamma_press_te288_128
  mhz_662d.basis'
hzpppm= 1.2776e+02
icolen= 10
icolst= 5
irowen= 13
```

P19 - tumor
voxel 12;7

Row#12 Col#7 MR Hirn (2019.01.09 17:36:15) Subject 10 7.86e-01 mL, TE/TR/NS=288/1500/3 (s2DSI_TE288 TR1500)

Data of: Sektion Biomedizinische Bildgebung, Christian-Albrechts-Universitaet zu Kiel

LCModel (Version 6.3-1J) Copyright: S.W. Provencher. *Ref.: Magn. Reson. Med. 30:672-679 (1993).* *14-June-2019 17:23*

Conc.	%SD	/GPC+PC	Metabolite
**88.337**	**14%**	**3.139**	**Ala**
20.430	42%	0.726	Cr
0.000	999%	0.000	PCr
0.000	999%	0.000	Gln
0.000	999%	0.000	Glu
0.000	999%	0.000	GPC
**28.142**	**11%**	**1.000**	**PCh**
5.852	160%	0.208	GSH
0.000	999%	0.000	Ins
**152.662**	**8%**	**5.425**	**Lac**
29.949	29%	1.064	NAA
3.323	127%	0.118	NAAG
1.650	74%	5.9E-02	Scyllo
5.733	107%	0.204	Tau
0.000	999%	0.000	-CrCH2
**28.142**	**11%**	**1.000**	**GPC+PCh**
33.272	27%	1.182	NAA+NAAG
20.430	42%	0.726	Cr+PCr
0.000	999%	0.000	Glu+Gln
0.000	999%	0.000	Lip13a
0.000	999%	0.000	Lip13b
0.000	999%	0.000	Lip20
0.000	999%	0.000	Lip13a+Lip13b

DIAGNOSTICS

```
1 info      STARTV 24
6 info's    RFALSI 11
4 info's    RFALSI  4
2 info's    TWOREG  8
```

MISCELLANEOUS OUTPUT

```
FWHM = 0.138 ppm    S/N =   2
Data shift = 0.054 ppm
Ph: -17 deg      -16.1 deg/ppm
```

INPUT CHANGES

```
deltat= 5.000e-04
echot= 288.00
filbas= '/home/stu111733/.lcmodel/basis-sets/gam
  ma_press_te288_128mhz_662d.basis'
hzpppm= 1.2776e+02
icolen= 10
icolst= 5
irowen= 13
irowst= 9
islice= 1
lps= 8
ltable= 7
ndcols= 11
ndrows= 14
ndslic= 1
nunfil= 1024
ppmend= 1.0
ppmst= 4.0
savdir= '/home/data/Studies/GBM_Spektro/results/
  '
sptype= 'tumor'
srcraw= '/home/data/Studies/GBM_Spektro/data/
ste288_5_1_raw_act.SDAT'
```

P19 - edema
voxel 10;10

Row#10 Col#10 MR Hirn (2019.01.09 17:36:15) Subject 10 7.86e-01 mL, TE/TR/NS=288/1500/3 (s2DSI_TE288 TR1500)

Data of: Sektion Biomedizinische Bildgebung, Christian-Albrechts-Universitaet zu Kiel

LCModel (Version 6.3-1J) Copyright: S.W. Provencher.   Ref.: Magn. Reson. Med. 30:672-679 (1993).   *14-June-2019 17:24*

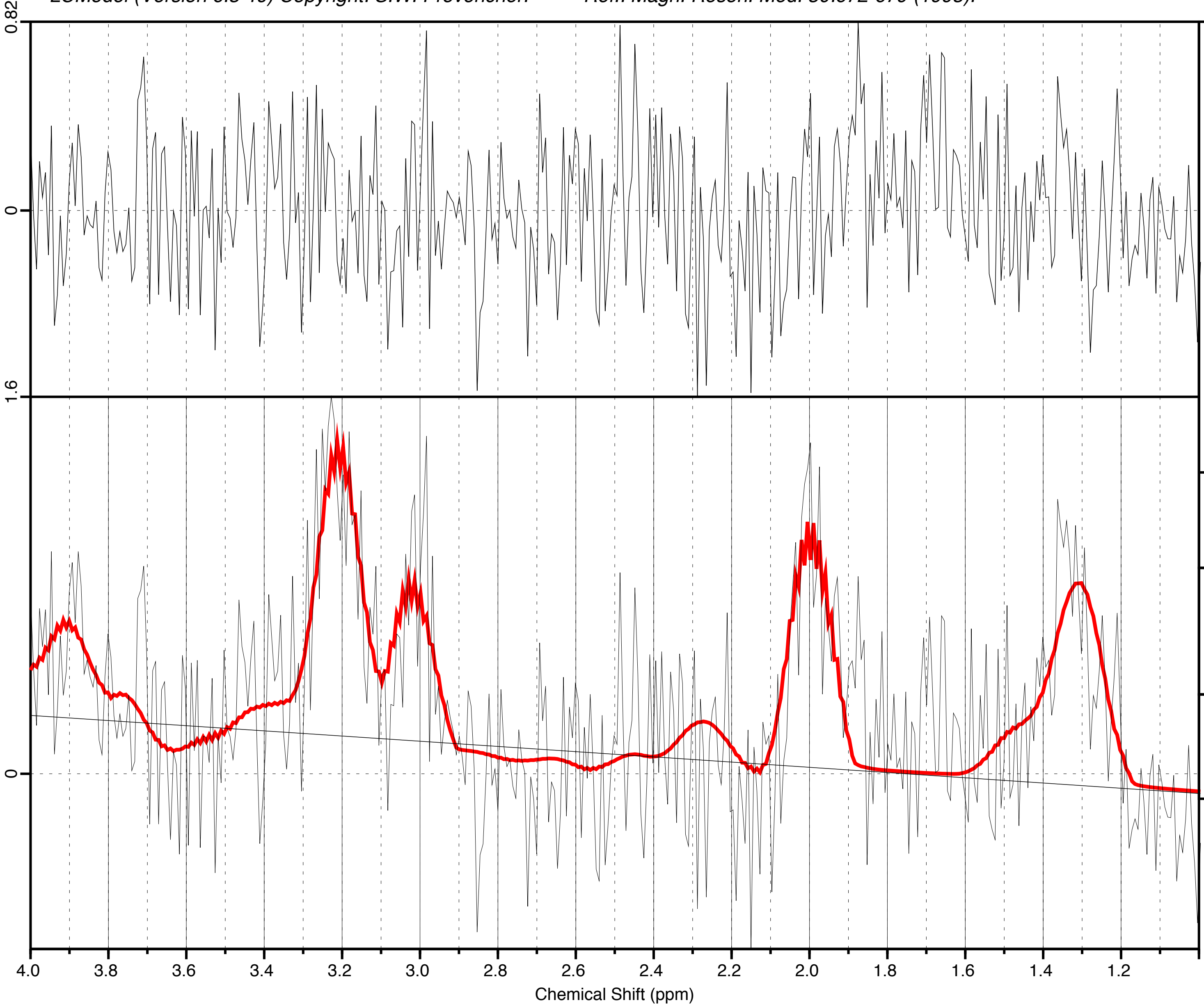

Conc.	%SD	/GPC+PC	Metabolite
18.625	48%	0.746	Ala
40.305	114%	1.615	Cr
3.153	999%	0.126	PCr
0.000	999%	0.000	Gln
35.840	52%	1.436	Glu
15.868	83%	0.636	GPC
9.092	148%	0.364	PCh
0.000	999%	0.000	GSH
43.175	180%	1.730	Ins
**62.101**	**12%**	**2.488**	**Lac**
**63.824**	**11%**	**2.557**	**NAA**
0.000	999%	0.000	NAAG
2.347	70%	9.4E-02	Scyllo
13.241	63%	0.530	Tau
6.600	119%	0.264	-CrCH2
**24.960**	**10%**	**1.000**	**GPC+PCh**
**63.824**	**11%**	**2.557**	**NAA+NAAG**
43.458	16%	1.741	Cr+PCr
35.840	52%	1.436	Glu+Gln
0.000	999%	0.000	Lip13a
0.000	999%	0.000	Lip13b
0.000	999%	0.000	Lip20
0.000	999%	0.000	Lip13a+Lip13b

DIAGNOSTICS

1 info   STARTV 24
7 info's RFALSI 11
3 info's RFALSI 4
1 info   TWOREG 8

MISCELLANEOUS OUTPUT

FWHM = 0.122 ppm   S/N = 2
Data shift = 0.046 ppm
Ph: 12 deg   -7.0 deg/ppm

INPUT CHANGES

deltat= 5.000e-04
echot= 288.00
filbas= '/home/stu111733/.lcmodel/basis-sets/gamma_press_te288_128mhz_662d.basis'
hzpppm= 1.2776e+02
icolen= 10
icolst= 5
irowen= 13

P19 - edema
voxel 10;10

Row#10 Col#10 MR Hirn (2019.01.09 17:36:15) Subject 10 7.86e-01 mL, TE/TR/NS=288/1500/3 (s2DSI_TE288 TR1500)

Data of: Sektion Biomedizinische Bildgebung, Christian-Albrechts-Universitaet zu Kiel

LCModel (Version 6.3-1J) Copyright: S.W. Provencher. *Ref.: Magn. Reson. Med. 30:672-679 (1993).* *14-June-2019 17:24*

Conc.	%SD	/GPC+PC	Metabolite
18.625	48%	0.746	Ala
40.305	114%	1.615	Cr
3.153	999%	0.126	PCr
0.000	999%	0.000	Gln
35.840	52%	1.436	Glu
15.868	83%	0.636	GPC
9.092	148%	0.364	PCh
0.000	999%	0.000	GSH
43.175	180%	1.730	Ins
**62.101**	**12%**	**2.488**	**Lac**
**63.824**	**11%**	**2.557**	**NAA**
0.000	999%	0.000	NAAG
2.347	70%	9.4E-02	Scyllo
13.241	63%	0.530	Tau
6.600	119%	0.264	-CrCH2
**24.960**	**10%**	**1.000**	**GPC+PCh**
**63.824**	**11%**	**2.557**	**NAA+NAAG**
43.458	16%	1.741	Cr+PCr
35.840	52%	1.436	Glu+Gln
0.000	999%	0.000	Lip13a
0.000	999%	0.000	Lip13b
0.000	999%	0.000	Lip20
0.000	999%	0.000	Lip13a+Lip13b

DIAGNOSTICS

```
1 info      STARTV 24
7 info's    RFALSI 11
3 info's    RFALSI  4
1 info      TWOREG  8
```

MISCELLANEOUS OUTPUT

```
FWHM = 0.122 ppm    S/N =   2
Data shift = 0.046 ppm
Ph:  12 deg      -7.0 deg/ppm
```

INPUT CHANGES

```
deltat= 5.000e-04
echot= 288.00
filbas= '/home/stu111733/.lcmodel/basis-sets/gam
  ma_press_te288_128mhz_662d.basis'
hzpppm= 1.2776e+02
icolen= 10
icolst= 5
irowen= 13
irowst= 9
islice= 1
lps= 8
ltable= 7
ndcols= 11
ndrows= 14
ndslic= 1
nunfil= 1024
ppmend= 1.0
ppmst= 4.0
savdir= '/home/data/Studies/GBM_Spektro/results/
  '
sptype= 'tumor'
srcraw= '/home/data/Studies/GBM_Spektro/data/
site288_5_1_raw_act.SDAT'
```

P30 - tumor
voxel 11;4

Row#11 Col#4 MR Spektroskopie (2019.08.27 10:25:40) Subject 04 8.00e-01 mL, TE/TR/NS=288/1500/2 (s2DSI_TE288 TR1500)

Data of: Sektion Biomedizinische Bildgebung, Christian-Albrechts-Universitaet zu Kiel

LCModel (Version 6.3-1J) Copyright: S.W. Provencher. *Ref.: Magn. Reson. Med. 30:672-679 (1993).* *28-November-2019 17:11*

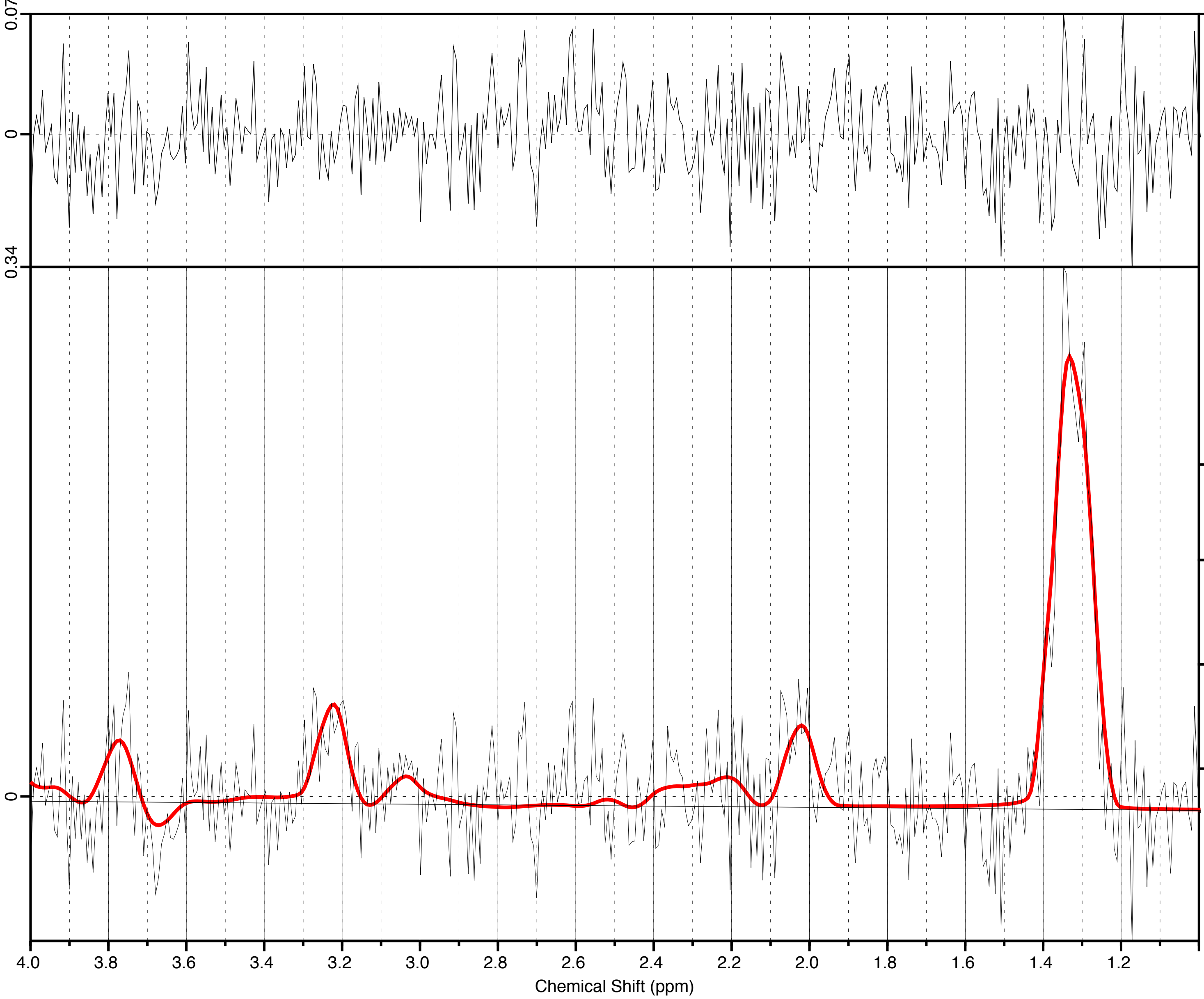

Conc.	%SD	/GPC+PC	Metabolite
0.000	999%	0.000	Ala
0.000	999%	0.000	Cr
0.778	59%	0.873	PCr
1.960	113%	2.198	Gln
1.381	138%	1.549	Glu
0.892	18%	1.000	GPC
0.000	999%	0.000	PCh
1.088	61%	1.220	GSH
0.000	999%	0.000	Ins
**16.384**	**4%**	**18.370**	**Lac**
2.012	23%	2.257	NAA
0.357	48%	0.401	NAAG
4.69E-02	114%	5.3E-02	Scyllo
0.305	87%	0.342	Tau
9.86E-02	199%	0.111	-CrCH2
0.892	18%	1.000	GPC+PCh
2.370	21%	2.657	NAA+NAAG
0.778	59%	0.873	Cr+PCr
3.341	51%	3.746	Glu+Gln
0.000	999%	0.000	Lip13a
0.000	999%	0.000	Lip13b
2.40E-06	999%	2.7E-06	Lip20
0.000	999%	0.000	Lip13a+Lip13b

DIAGNOSTICS

7 info's	RFALSI 11
3 info's	RFALSI 4
1 info	TWOREG 8

MISCELLANEOUS OUTPUT

FWHM = 0.076 ppm S/N = 5
Data shift = 0.023 ppm
Ph: 29 deg 12.6 deg/ppm

INPUT CHANGES

deltat= 5.000e-04
echot= 288.00
filbas= '/home/stu111733/.lcmodel/basis-sets/gamma_press_te288_128mhz_662d.basis'
hzpppm= 1.2776e+02
icolen= 7
icolst= 3
irowen= 15
irowst= 6

P30 - tumor
voxel 11;4

Row#11 Col#4 MR Spektroskopie (2019.08.27 10:25:40) Subject 04 8.00e-01 mL, TE/TR/NS=288/1500/2 (s2DSI_TE288 TR1500)

Data of: Sektion Biomedizinische Bildgebung, Christian-Albrechts-Universitaet zu Kiel

LCModel (Version 6.3-1J) Copyright: S.W. Provencher. *Ref.: Magn. Reson. Med. 30:672-679 (1993).* *28-November-2019 17:11*

Conc.	%SD	/GPC+PC	Metabolite
0.000	999%	0.000	Ala
0.000	999%	0.000	Cr
0.778	59%	0.873	PCr
1.960	113%	2.198	Gln
1.381	138%	1.549	Glu
0.892	18%	1.000	GPC
0.000	999%	0.000	PCh
1.088	61%	1.220	GSH
0.000	999%	0.000	Ins
**16.384**	**4%**	**18.370**	**Lac**
2.012	23%	2.257	NAA
0.357	48%	0.401	NAAG
4.69E-02	114%	5.3E-02	Scyllo
0.305	87%	0.342	Tau
9.86E-02	199%	0.111	-CrCH2
0.892	18%	1.000	GPC+PCh
2.370	21%	2.657	NAA+NAAG
0.778	59%	0.873	Cr+PCr
3.341	51%	3.746	Glu+Gln
0.000	999%	0.000	Lip13a
0.000	999%	0.000	Lip13b
2.40E-06	999%	2.7E-06	Lip20
0.000	999%	0.000	Lip13a+Lip13b

DIAGNOSTICS

```
7 info's    RFALSI 11
3 info's    RFALSI  4
1 info      TWOREG  8
```

MISCELLANEOUS OUTPUT

```
FWHM = 0.076 ppm    S/N =   5
Data shift = 0.023 ppm
Ph:  29 deg       12.6 deg/ppm
```

INPUT CHANGES

```
deltat= 5.000e-04
echot= 288.00
filbas= '/home/stu111733/.lcmodel/basis-sets/gam
  ma_press_te288_128mhz_662d.basis'
hzpppm= 1.2776e+02
icolen= 7
icolst= 3
irowen= 15
irowst= 6
islice= 1
lps= 8
ltable= 7
ndcols= 16
ndrows= 20
ndslic= 1
nunfil= 1024
ppmend= 1.0
ppmst= 4.0
savdir= '/home/stu111733/.lcmodel/saved/MR_Spekt
  roskopie/s04/2019.08.27_10:25:40    /'
sptype= 'tumor'
srcraw= '/home/data/Studies/GBM_Spektro/data/s04

/DBIEX_5_1_raw_act.SDAT'
```

P30 - edema
voxel 14;4

Row#11 Col#4 MR Spektroskopie (2019.08.27 10:25:40) Subject 04 8.00e-01 mL, TE/TR/NS=288/1500/2 (s2DSI_TE288 TR1500)

Data of: Sektion Biomedizinische Bildgebung, Christian-Albrechts-Universitaet zu Kiel

LCModel (Version 6.3-1J) Copyright: S.W. Provencher. Ref.: Magn. Reson. Med. 30:672-679 (1993). *28-November-2019 17:11*

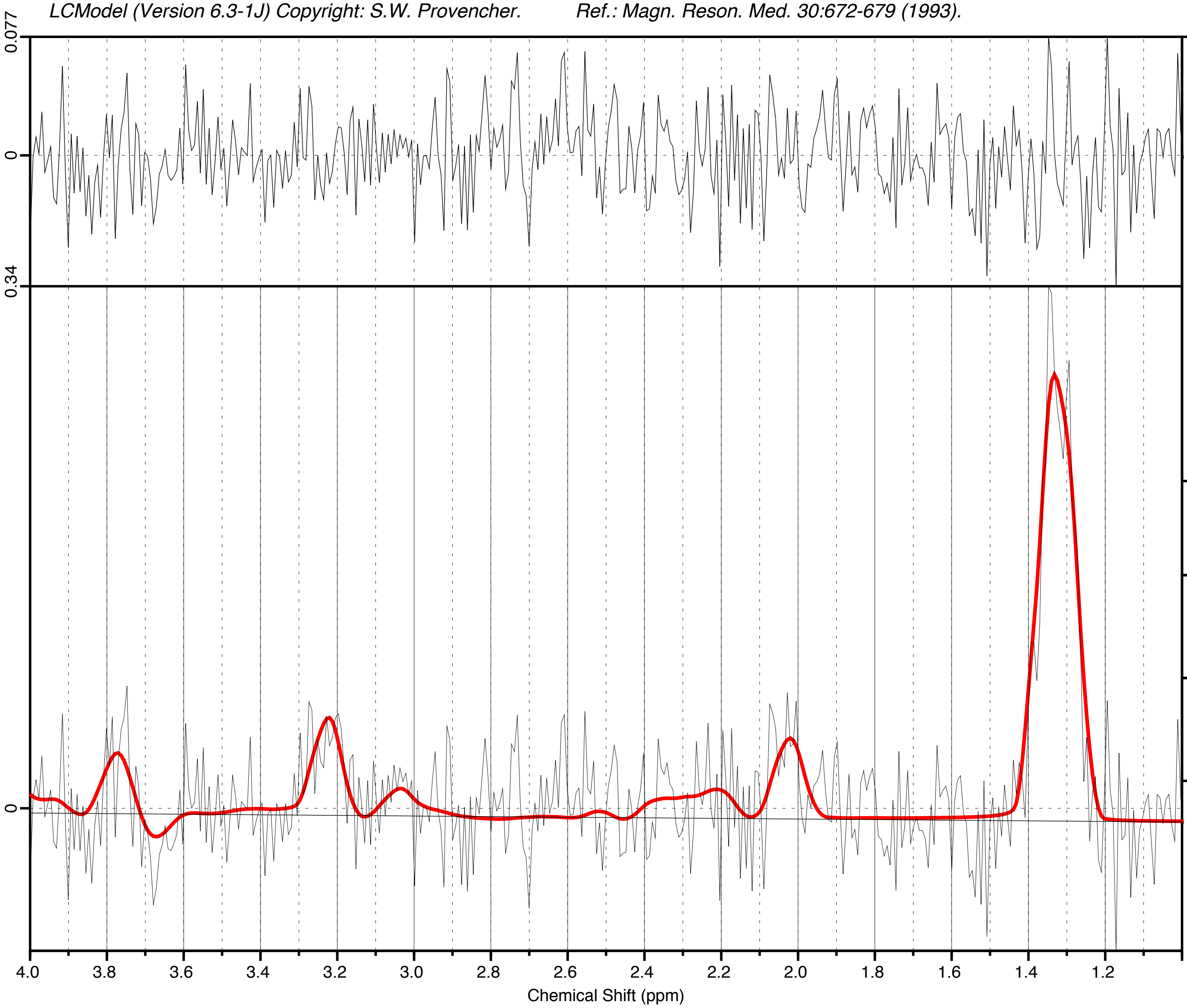

Conc.	%SD	/GPC+PC	Metabolite
0.000	999%	0.000	Ala
0.000	999%	0.000	Cr
0.778	59%	0.873	PCr
1.960	113%	2.198	Gln
1.381	138%	1.549	Glu
0.892	18%	1.000	GPC
0.000	999%	0.000	PCh
1.088	61%	1.220	GSH
0.000	999%	0.000	Ins
16.384	4%	18.370	Lac
2.012	23%	2.257	NAA
0.357	48%	0.401	NAAG
4.69E-02	114%	5.3E-02	Scyllo
0.305	87%	0.342	Tau
9.86E-02	199%	0.111	-CrCH2
0.892	18%	1.000	GPC+PCh
2.370	21%	2.657	NAA+NAAG
0.778	59%	0.873	Cr+PCr
3.341	51%	3.746	Glu+Gln
0.000	999%	0.000	Lip13a
0.000	999%	0.000	Lip13b
2.40E-06	999%	2.7E-06	Lip20
0.000	999%	0.000	Lip13a+Lip13b

DIAGNOSTICS

7 info's	RFALSI	11
3 info's	RFALSI	4
1 info	TWOREG	8

MISCELLANEOUS OUTPUT

FWHM = 0.076 ppm S/N = 5
Data shift = 0.023 ppm
Ph: 29 deg 12.6 deg/ppm

INPUT CHANGES

deltat= 5.000e-04
echot= 288.00
filbas= '/home/stu111733/.lcmodel/basis-sets/gamma_press_te288_128mhz_662d.basis'
hzpppm= 1.2776e+02
icolen= 7
icolst= 3
irowen= 15
irowst= 6

P30 - edema
voxel 14;4

Row#11 Col#4 MR Spektroskopie (2019.08.27 10:25:40) Subject 04 8.00e-01 mL, TE/TR/NS=288/1500/2 (s2DSI_TE288 TR1500)

Data of: Sektion Biomedizinische Bildgebung, Christian-Albrechts-Universitaet zu Kiel

LCModel (Version 6.3-1J) Copyright: S.W. Provencher. *Ref.: Magn. Reson. Med. 30:672-679 (1993).* *28-November-2019 17:11*

Conc.	%SD	/GPC+PC	Metabolite
0.000	999%	0.000	Ala
0.000	999%	0.000	Cr
0.778	59%	0.873	PCr
1.960	113%	2.198	Gln
1.381	138%	1.549	Glu
0.892	18%	1.000	GPC
0.000	999%	0.000	PCh
1.088	61%	1.220	GSH
0.000	999%	0.000	Ins
**16.384**	**4%**	**18.370**	**Lac**
2.012	23%	2.257	NAA
0.357	48%	0.401	NAAG
4.69E-02	114%	5.3E-02	Scyllo
0.305	87%	0.342	Tau
9.86E-02	199%	0.111	-CrCH2
0.892	18%	1.000	GPC+PCh
2.370	21%	2.657	NAA+NAAG
0.778	59%	0.873	Cr+PCr
3.341	51%	3.746	Glu+Gln

0.000	999%	0.000	Lip13a
0.000	999%	0.000	Lip13b
2.40E-06	999%	2.7E-06	Lip20
0.000	999%	0.000	Lip13a+Lip13b

DIAGNOSTICS

7 info's	RFALSI	11
3 info's	RFALSI	4
1 info	TWOREG	8

MISCELLANEOUS OUTPUT

FWHM = 0.076 ppm   S/N = 5
Data shift = 0.023 ppm
Ph: 29 deg   12.6 deg/ppm

INPUT CHANGES

deltat= 5.000e-04
echot= 288.00
filbas= '/home/stu111733/.lcmodel/basis-sets/gamma_press_te288_128mhz_662d.basis'
hzpppm= 1.2776e+02
icolen= 7
icolst= 3
irowen= 15
irowst= 6
islice= 1
lps= 8
ltable= 7
ndcols= 16
ndrows= 20
ndslic= 1
nunfil= 1024
ppmend= 1.0
ppmst= 4.0
savdir= '/home/stu111733/.lcmodel/saved/MR_Spektroskopie/s04/2019.08.27_10:25:40 /'
sptype= 'tumor'
srcraw= '/home/data/Studies/GBM_Spektro/data/s04

/DBIEX_5_1_raw_act.SDAT'

P42 - tumor
voxel 16;7

Row#16 Col#7 (2019.08.27 10:55:20) Subject 11 7.88e-01 mL, TE/TR/NS=288/1500/2 (s2DSI_TE288 TR1500)

Data of: Sektion Biomedizinische Bildgebung, Christian-Albrechts-Universitaet zu Kiel

LCModel (Version 6.3-1J) Copyright: S.W. Provencher. *Ref.: Magn. Reson. Med. 30:672-679 (1993).* *28-November-2019 16:41*

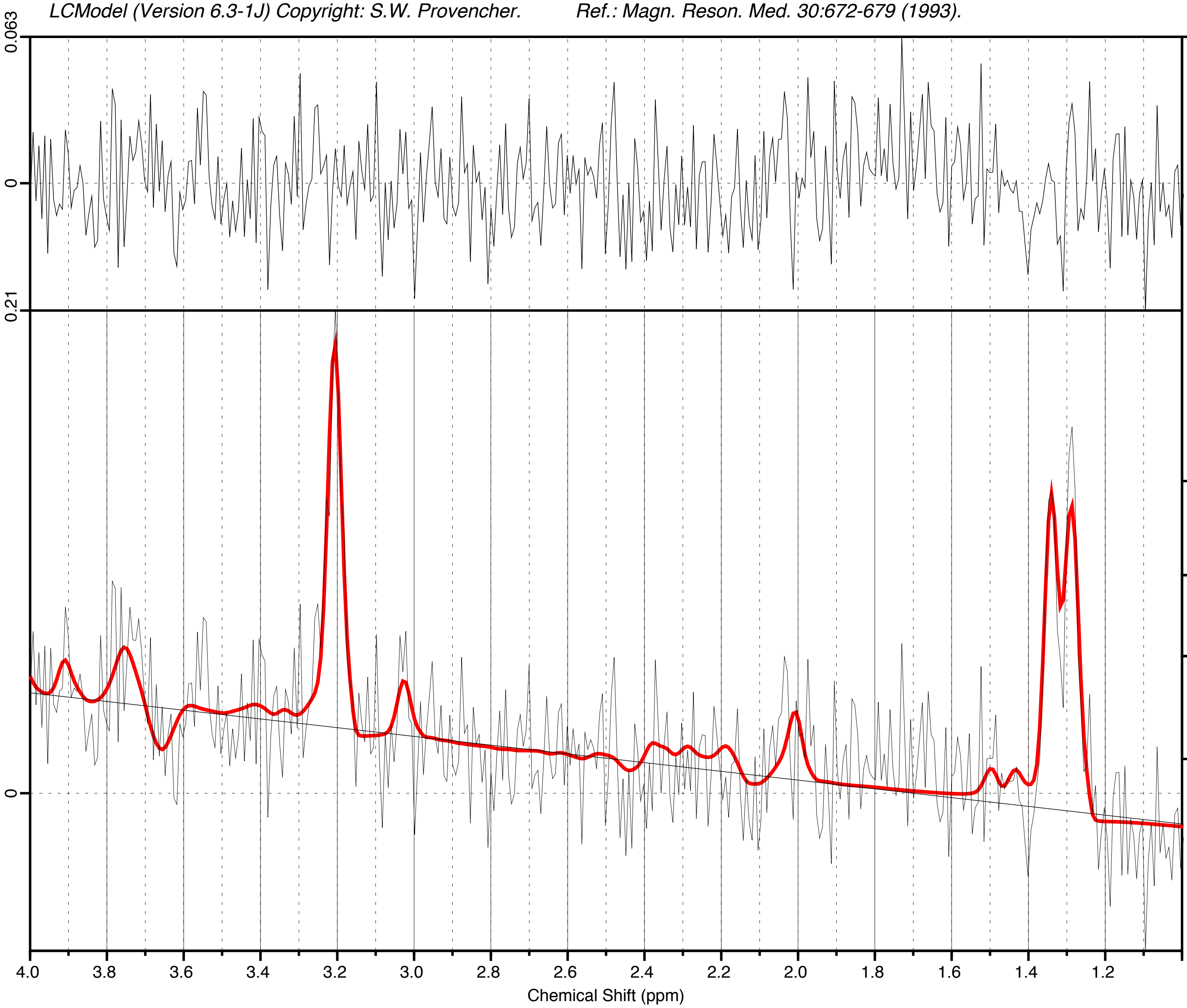

Conc.	%SD	/GPC+PC	Metabolite
0.600	74%	0.458	Ala
0.595	40%	0.454	Cr
0.000	999%	0.000	PCr
1.236	79%	0.944	Gln
0.702	139%	0.536	Glu
0.887	63%	0.677	GPC
0.424	134%	0.323	PCh
0.216	157%	0.165	GSH
0.000	999%	0.000	Ins
**6.092**	**6%**	**4.650**	**Lac**
0.669	37%	0.510	NAA
0.111	48%	8.5E-02	NAAG
4.57E-02	114%	3.5E-02	Scyllo
0.378	68%	0.288	Tau
9.80E-02	181%	7.5E-02	-CrCH2
**1.310**	**8%**	**1.000**	**GPC+PCh**
0.780	37%	0.595	NAA+NAAG
0.595	40%	0.454	Cr+PCr
1.939	50%	1.480	Glu+Gln
0.000	999%	0.000	Lip13a
0.000	999%	0.000	Lip13b
0.000	999%	0.000	Lip20
0.000	999%	0.000	Lip13a+Lip13b

DIAGNOSTICS

4 info's RFALSI 11
2 info's RFALSI 4

MISCELLANEOUS OUTPUT

FWHM = 0.038 ppm S/N = 4
Data shift = 0.008 ppm
Ph: 0 deg 0.2 deg/ppm

INPUT CHANGES

deltat= 5.000e-04
echot= 288.00
filbas= '/home/stu111733/.lcmodel/basis-sets/gamma_press_te288_128mhz_662d.basis'
hzpppm= 1.2776e+02
icolen= 8
icolst= 3
irowen= 17
irowst= 10
islice= 1

P42 - tumor
voxel 16;7

Row#16 Col#7 (2019.08.27 10:55:20) Subject 11 7.88e-01 mL, TE/TR/NS=288/1500/2 (s2DSI_TE288 TR1500)

Data of: Sektion Biomedizinische Bildgebung, Christian-Albrechts-Universitaet zu Kiel

LCModel (Version 6.3-1J) Copyright: S.W. Provencher. *Ref.: Magn. Reson. Med. 30:672-679 (1993).*

28-November-2019 16:41

Conc.	%SD	/GPC+PC	Metabolite
0.600	74%	0.458	Ala
0.595	40%	0.454	Cr
0.000	999%	0.000	PCr
1.236	79%	0.944	Gln
0.702	139%	0.536	Glu
0.887	63%	0.677	GPC
0.424	134%	0.323	PCh
0.216	157%	0.165	GSH
0.000	999%	0.000	Ins
**6.092**	**6%**	**4.650**	**Lac**
0.669	37%	0.510	NAA
0.111	48%	8.5E-02	NAAG
4.57E-02	114%	3.5E-02	Scyllo
0.378	68%	0.288	Tau
9.80E-02	181%	7.5E-02	-CrCH2
**1.310**	**8%**	**1.000**	**GPC+PCh**
0.780	37%	0.595	NAA+NAAG
0.595	40%	0.454	Cr+PCr
1.939	50%	1.480	Glu+Gln
0.000	999%	0.000	Lip13a
0.000	999%	0.000	Lip13b
0.000	999%	0.000	Lip20
0.000	999%	0.000	Lip13a+Lip13b

DIAGNOSTICS

```
4 info's    RFALSI 11
2 info's    RFALSI  4
```

MISCELLANEOUS OUTPUT

```
FWHM = 0.038 ppm    S/N =   4
Data shift = 0.008 ppm
Ph:   0 deg        0.2 deg/ppm
```

INPUT CHANGES

```
deltat= 5.000e-04
echot= 288.00
filbas= '/home/stu111733/.lcmodel/basis-sets/gam
  ma_press_te288_128mhz_662d.basis'
hzpppm= 1.2776e+02
icolen= 8
icolst= 3
irowen= 17
irowst= 10
```

```
islice= 1
lps= 8
ltable= 7
ndcols= 16
ndrows= 20
ndslic= 1
nunfil= 1024
ppmend= 1.0
ppmst= 4.0
savdir= '/home/stu111733/.lcmodel/saved/'
sptype= 'tumor'
srcraw= '/home/data/Studies/GBM_Spektro/data/s11
/DBIEX_5_1_raw_act.SDAT'
```

P42 - edema
voxel 13;6

Row#13 Col#6 (2019.08.27 10:55:20) Subject 11 7.88e-01 mL, TE/TR/NS=288/1500/2 (s2DSI_TE288 TR1500)

Data of: Sektion Biomedizinische Bildgebung, Christian-Albrechts-Universitaet zu Kiel

LCModel (Version 6.3-1J) Copyright: S.W. Provencher.   Ref.: Magn. Reson. Med. 30:672-679 (1993).   *28-November-2019 16:40*

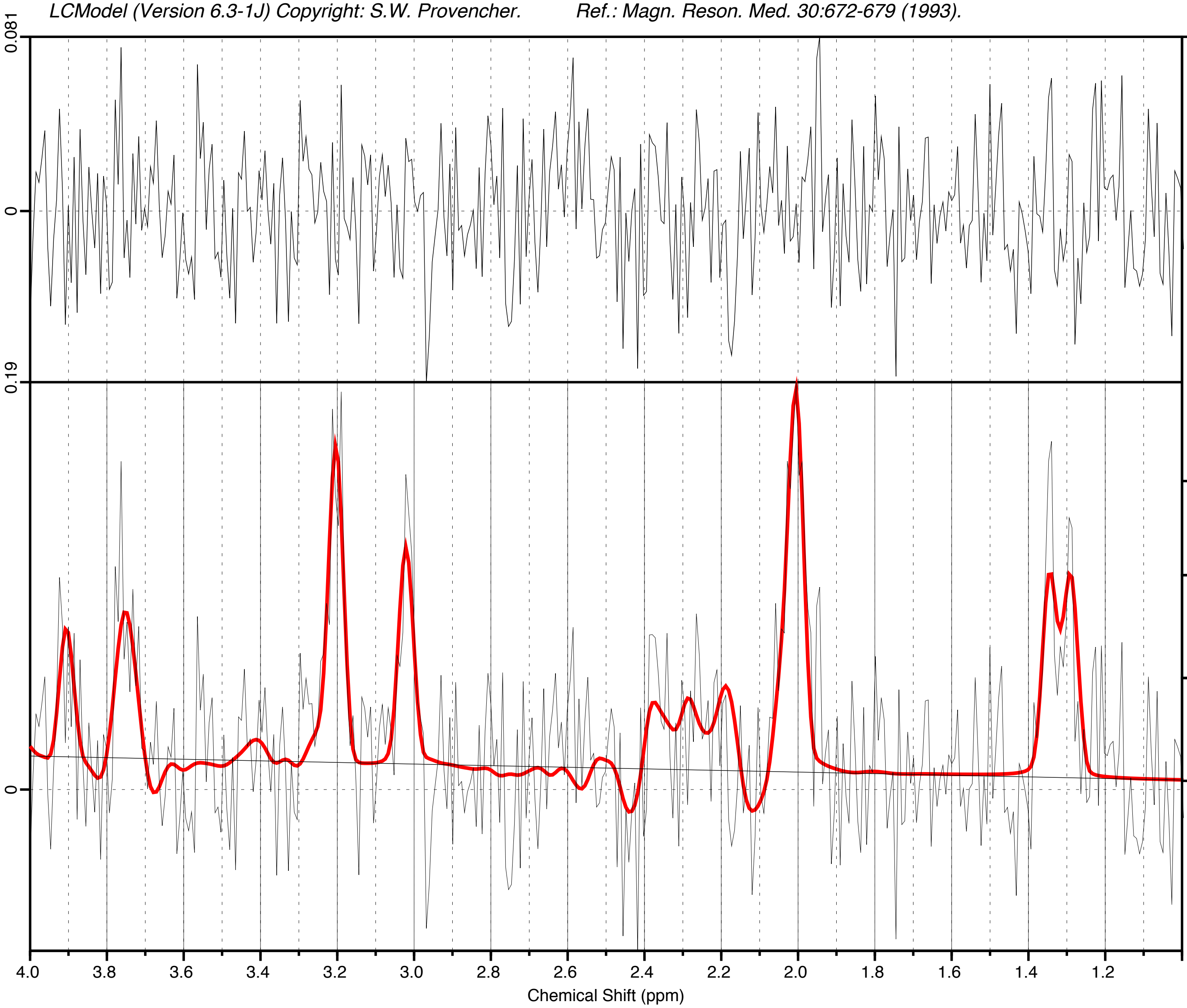

Conc.	%SD	/GPC+PC	Metabolite
0.000	999%	0.000	Ala
2.388	41%	2.034	Cr
7.26E-02	999%	6.2E-02	PCr
5.050	29%	4.301	Gln
2.632	54%	2.242	Glu
0.000	999%	0.000	GPC
**1.174**	**12%**	**1.000**	**PCh**
0.291	174%	0.248	GSH
0.000	999%	0.000	Ins
4.465	18%	3.804	Lac
**4.134**	**11%**	**3.521**	**NAA**
1.006	38%	0.857	NAAG
5.77E-02	170%	4.9E-02	Scyllo
0.931	52%	0.793	Tau
0.366	132%	0.312	-CrCH2
**1.174**	**12%**	**1.000**	**GPC+PCh**
**5.140**	**9%**	**4.378**	**NAA+NAAG**
2.461	15%	2.096	Cr+PCr
7.682	19%	6.543	Glu+Gln
0.153	999%	0.130	Lip13a
0.000	999%	0.000	Lip13b
2.29E-02	999%	2.0E-02	Lip20
0.153	999%	0.130	Lip13a+Lip13b

DIAGNOSTICS

12 info's	RFALSI 11
2 info's	RFALSI 4
1 info	RFALSI 12

MISCELLANEOUS OUTPUT

FWHM = 0.046 ppm   S/N =   3
Data shift = 0.000 ppm
Ph: -6 deg   2.1 deg/ppm

INPUT CHANGES

deltat= 5.000e-04
echot= 288.00
filbas= '/home/stu111733/.lcmodel/basis-sets/gamma_press_te288_128mhz_662d.basis'
hzpppm= 1.2776e+02
icolen= 8
icolst= 3
irowen= 17
irowst= 10

P42 - edema
voxel 13;6

Row#13 Col#6 (2019.08.27 10:55:20) Subject 11 7.88e-01 mL, TE/TR/NS=288/1500/2 (s2DSI_TE288 TR1500)

Data of: Sektion Biomedizinische Bildgebung, Christian-Albrechts-Universitaet zu Kiel

LCModel (Version 6.3-1J) Copyright: S.W. Provencher. *Ref.: Magn. Reson. Med. 30:672-679 (1993).*

28-November-2019 16:40

Conc.	%SD	/GPC+PC	Metabolite
0.000	999%	0.000	Ala
2.388	41%	2.034	Cr
7.26E-02	999%	6.2E-02	PCr
5.050	29%	4.301	Gln
2.632	54%	2.242	Glu
0.000	999%	0.000	GPC
**1.174**	**12%**	**1.000**	**PCh**
0.291	174%	0.248	GSH
0.000	999%	0.000	Ins
4.465	18%	3.804	Lac
**4.134**	**11%**	**3.521**	**NAA**
1.006	38%	0.857	NAAG
5.77E-02	170%	4.9E-02	Scyllo
0.931	52%	0.793	Tau
0.366	132%	0.312	-CrCH2
**1.174**	**12%**	**1.000**	**GPC+PCh**
**5.140**	**9%**	**4.378**	**NAA+NAAG**
2.461	15%	2.096	Cr+PCr
7.682	19%	6.543	Glu+Gln
0.153	999%	0.130	Lip13a
0.000	999%	0.000	Lip13b
2.29E-02	999%	2.0E-02	Lip20
0.153	999%	0.130	Lip13a+Lip13b

DIAGNOSTICS

```
 12 info's    RFALSI 11
  2 info's    RFALSI  4
  1 info      RFALSI 12
```

MISCELLANEOUS OUTPUT

```
FWHM = 0.046 ppm    S/N =   3
Data shift = 0.000 ppm
Ph:  -6 deg        2.1 deg/ppm
```

INPUT CHANGES

```
deltat= 5.000e-04
echot= 288.00
filbas= '/home/stu111733/.lcmodel/basis-sets/gam
  ma_press_te288_128mhz_662d.basis'
hzpppm= 1.2776e+02
icolen= 8
icolst= 3
irowen= 17
irowst= 10
islice= 1
lps= 8
ltable= 7
ndcols= 16
ndrows= 20
ndslic= 1
nunfil= 1024
ppmend= 1.0
ppmst= 4.0
savdir= '/home/stu111733/.lcmodel/saved/'
sptype= 'tumor'
srcraw= '/home/data/Studies/GBM_Spektro/data/s11
/DBIEX_5_1_raw_act.SDAT'
```

P43 - tumor
voxel 14;11

Row#14 Col#11 MR Hirn funktionell (2019.04.26 14:02:53) Subject 12 7.99e-01 mL, TE/TR/NS=288/1500/2 (s2DSI_TE288 TR1500)

Data of: Sektion Biomedizinische Bildgebung, Christian-Albrechts-Universitaet zu Kiel

LCModel (Version 6.3-1J) Copyright: S.W. Provencher. Ref.: Magn. Reson. Med. 30:672-679 (1993). *17-June-2019 14:55*

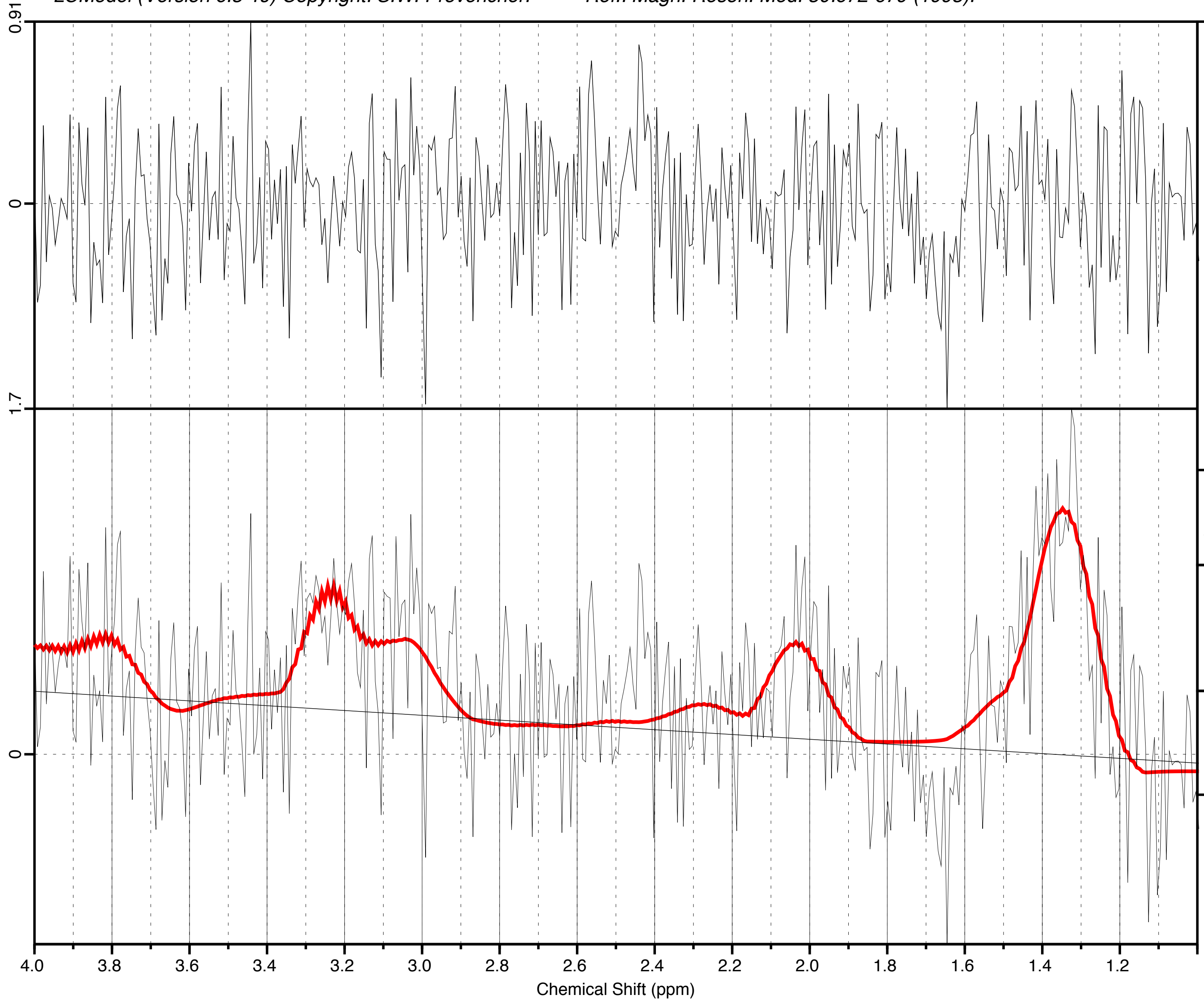

Conc.	%SD	/GPC+PC	Metabolite
21.419	60%	1.425	Ala
29.821	29%	1.984	Cr
0.000	999%	0.000	PCr
0.000	999%	0.000	Gln
24.580	101%	1.635	Glu
15.029	19%	1.000	GPC
0.000	999%	0.000	PCh
13.305	73%	0.885	GSH
0.000	999%	0.000	Ins
104.734	10%	6.969	Lac
33.622	23%	2.237	NAA
5.187	49%	0.345	NAAG
1.190	85%	7.9E-02	Scyllo
5.781	88%	0.385	Tau
8.622	90%	0.574	-CrCH2
15.029	19%	1.000	GPC+PCh
38.810	22%	2.582	NAA+NAAG
29.821	29%	1.984	Cr+PCr
24.580	101%	1.635	Glu+Gln
0.000	999%	0.000	Lip13a
0.000	999%	0.000	Lip13b
3.82E-05	999%	2.5E-06	Lip20
0.000	999%	0.000	Lip13a+Lip13b

DIAGNOSTICS

1 warning	STARTV 25
1 info	STARTV 24
8 info's	RFALSI 11
2 info's	RFALSI 4

MISCELLANEOUS OUTPUT

FWHM = 0.168 ppm S/N = 2
Data shift = 0.054 ppm
Ph: 7 deg -6.7 deg/ppm

INPUT CHANGES

deltat= 5.000e-04
echot= 288.00
filbas= '/home/stu111733/.lcmodel/basis-sets/gamma_press_te288_128mhz_662d.basis'
hzpppm= 1.2776e+02
icolen= 13
icolst= 11
irowen= 14

P43 - tumor
voxel 14;11

Row#14 Col#11 MR Hirn funktionell (2019.04.26 14:02:53) Subject 12 7.99e-01 mL, TE/TR/NS=288/1500/2 (s2DSI_TE288 TR1500)

Data of: Sektion Biomedizinische Bildgebung, Christian-Albrechts-Universitaet zu Kiel

LCModel (Version 6.3-1J) Copyright: S.W. Provencher. *Ref.: Magn. Reson. Med. 30:672-679 (1993).* *17-June-2019 14:55*

Conc.	%SD	/GPC+PC	Metabolite
21.419	60%	1.425	Ala
29.821	29%	1.984	Cr
0.000	999%	0.000	PCr
0.000	999%	0.000	Gln
24.580	101%	1.635	Glu
15.029	19%	1.000	GPC
0.000	999%	0.000	PCh
13.305	73%	0.885	GSH
0.000	999%	0.000	Ins
**104.734**	**10%**	**6.969**	**Lac**
33.622	23%	2.237	NAA
5.187	49%	0.345	NAAG
1.190	85%	7.9E-02	Scyllo
5.781	88%	0.385	Tau
8.622	90%	0.574	-CrCH2
15.029	19%	1.000	GPC+PCh
38.810	22%	2.582	NAA+NAAG
29.821	29%	1.984	Cr+PCr
24.580	101%	1.635	Glu+Gln
0.000	999%	0.000	Lip13a
0.000	999%	0.000	Lip13b
3.82E-05	999%	2.5E-06	Lip20
0.000	999%	0.000	Lip13a+Lip13b

DIAGNOSTICS

**1 warning**	**STARTV 25**
1 info	STARTV 24
8 info's	RFALSI 11
2 info's	RFALSI 4

MISCELLANEOUS OUTPUT

```
FWHM = 0.168 ppm     S/N =    2
Data shift = 0.054 ppm
Ph:    7 deg       -6.7 deg/ppm
```

INPUT CHANGES

```
deltat= 5.000e-04
echot= 288.00
filbas= '/home/stu111733/.lcmodel/basis-sets/gam
  ma_press_te288_128mhz_662d.basis'
hzpppm= 1.2776e+02
icolen= 13
icolst= 11
irowen= 14
irowst= 12
islice= 1
lps= 8
ltable= 7
ndcols= 16
ndrows= 20
ndslic= 1
nunfil= 1024
ppmend= 1.0
ppmst= 4.0
savdir= '/home/stu111733/.lcmodel/saved/'
sptype= 'tumor'
srcraw= '/home/data/Studies/GBM_Spektro/data/s12
/DBIEX_11_1_raw_act.SDAT'
```

P43 - edema
voxel 11;11

Row#11 Col#11 MR Hirn funktionell (2019.04.26 14:02:53) Subject 12 7.99e-01 mL, TE/TR/NS=288/1500/2 (s2DSI_TE288 TR1500)

Data of: Sektion Biomedizinische Bildgebung, Christian-Albrechts-Universitaet zu Kiel

LCModel (Version 6.3-1J) Copyright: S.W. Provencher. Ref.: Magn. Reson. Med. 30:672-679 (1993). *28-November-2019 15:28*

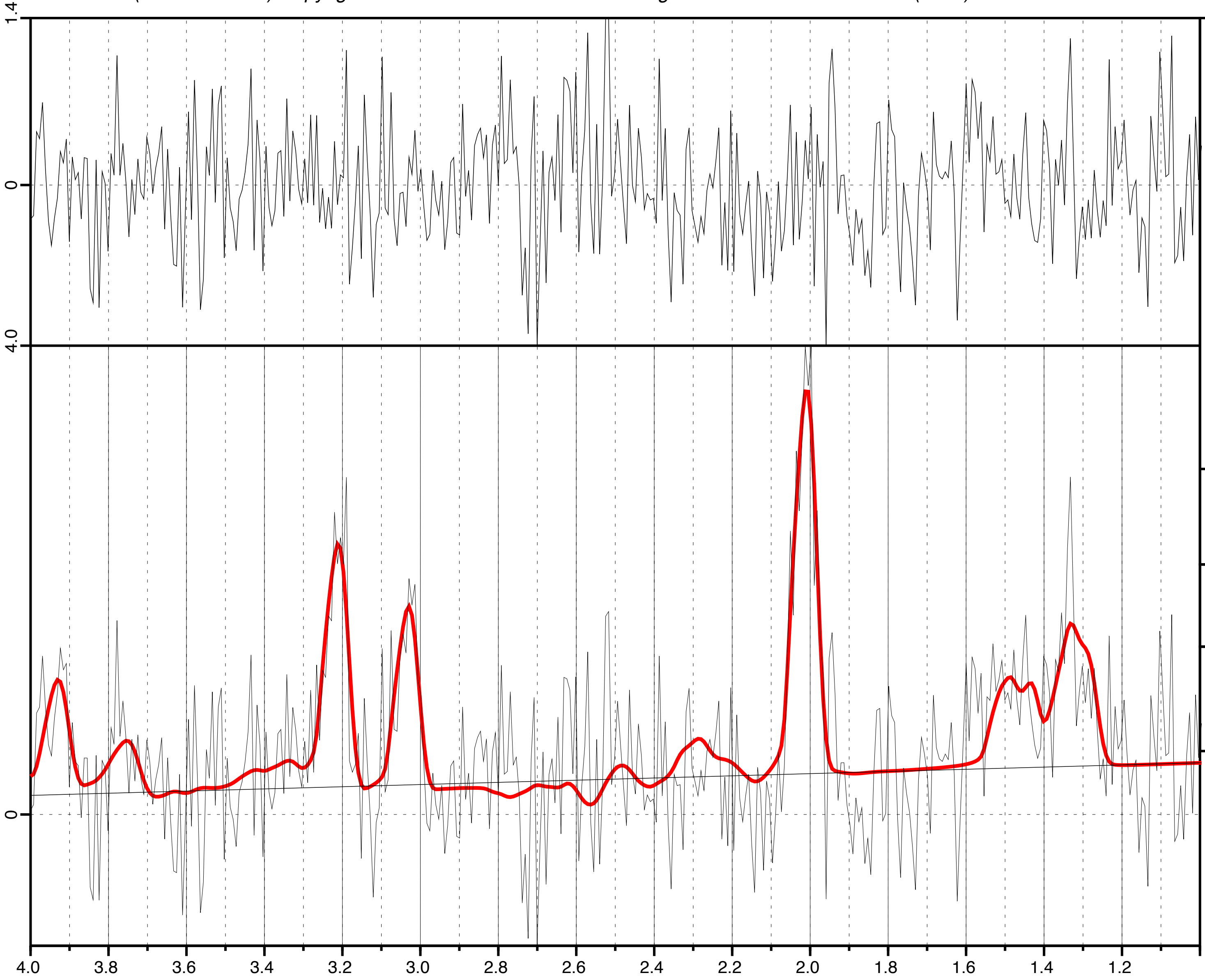

Conc.	%SD	/GPC+PC	Metabolite
50.804	23%	2.212	Ala
9.531	325%	0.415	Cr
43.349	72%	1.887	PCr
3.412	916%	0.149	Gln
41.767	69%	1.818	Glu
0.000	999%	0.000	GPC
**22.971**	**12%**	**1.000**	**PCh**
2.414	427%	0.105	GSH
0.000	999%	0.000	Ins
62.673	16%	2.728	Lac
**111.355**	**10%**	**4.848**	**NAA**
1.802	643%	7.8E-02	NAAG
3.512	61%	0.153	Scyllo
11.560	92%	0.503	Tau
3.593	278%	0.156	-CrCH2
**22.971**	**12%**	**1.000**	**GPC+PCh**
**113.157**	**8%**	**4.926**	**NAA+NAAG**
52.880	15%	2.302	Cr+PCr
45.179	61%	1.967	Glu+Gln
9.10E-05	999%	4.0E-06	Lip13a
0.000	999%	0.000	Lip13b
0.000	999%	0.000	Lip20
9.10E-05	999%	4.0E-06	Lip13a+Lip13b

DIAGNOSTICS

7 info's RFALSI 11
2 info's RFALSI 4

MISCELLANEOUS OUTPUT

FWHM = 0.061 ppm S/N = 3
Data shift = 0.015 ppm
Ph: -5 deg -8.5 deg/ppm

INPUT CHANGES

deltat= 5.000e-04
echot= 288.00
filbas= '/home/stu111733/.lcmodel/basis-sets/gamma_press_te288_128mhz_662d.basis'
hzpppm= 1.2776e+02
icolen= 13
icolst= 9
irowen= 11
irowst= 10
islice= 1

P43 - edema
voxel 11;11

Row#11 Col#11 MR Hirn funktionell (2019.04.26 14:02:53) Subject 12 7.99e-01 mL, TE/TR/NS=288/1500/2 (s2DSI_TE288 TR1500)

Data of: Sektion Biomedizinische Bildgebung, Christian-Albrechts-Universitaet zu Kiel

LCModel (Version 6.3-1J) Copyright: S.W. Provencher. *Ref.: Magn. Reson. Med. 30:672-679 (1993).* *28-November-2019 15:28*

Conc.	%SD	/GPC+PC	Metabolite
50.804	23%	2.212	Ala
9.531	325%	0.415	Cr
43.349	72%	1.887	PCr
3.412	916%	0.149	Gln
41.767	69%	1.818	Glu
0.000	999%	0.000	GPC
**22.971**	**12%**	**1.000**	**PCh**
2.414	427%	0.105	GSH
0.000	999%	0.000	Ins
62.673	16%	2.728	Lac
**111.355**	**10%**	**4.848**	**NAA**
1.802	643%	7.8E-02	NAAG
3.512	61%	0.153	Scyllo
11.560	92%	0.503	Tau
3.593	278%	0.156	-CrCH2
**22.971**	**12%**	**1.000**	**GPC+PCh**
**113.157**	**8%**	**4.926**	**NAA+NAAG**
52.880	15%	2.302	Cr+PCr
45.179	61%	1.967	Glu+Gln
9.10E-05	999%	4.0E-06	Lip13a
0.000	999%	0.000	Lip13b
0.000	999%	0.000	Lip20
9.10E-05	999%	4.0E-06	Lip13a+Lip13b

DIAGNOSTICS

7 info's RFALSI 11
2 info's RFALSI 4

MISCELLANEOUS OUTPUT

FWHM = 0.061 ppm S/N = 3
Data shift = 0.015 ppm
Ph: -5 deg -8.5 deg/ppm

INPUT CHANGES

deltat= 5.000e-04
echot= 288.00
filbas= '/home/stu111733/.lcmodel/basis-sets/gamma_press_te288_128mhz_662d.basis'
hzpppm= 1.2776e+02
icolen= 13
icolst= 9
irowen= 11
irowst= 10
islice= 1
lps= 8
ltable= 7
ndcols= 16
ndrows= 20
ndslic= 1
nunfil= 1024
ppmend= 1.0
ppmst= 4.0
savdir= '/home/stu111733/.lcmodel/saved/MR_Hirn_funktionell/s12/2019.04.26_14:02:53/' sptype= 'tumor'
srcraw= '/home/data/Studies/GBM_Spektro/data/s12ff/DBIEX_11_1_raw_act.SDAT'

P44 - tumor
voxel 11;5

Row#11 Col#5 MR Hirn (2018.11.14 09:25:43) Subject 14 8.00e-01 mL, TE/TR/NS=288/1500/1 (s2DSI_TE288 TR1500)

Data of: Sektion Biomedizinische Bildgebung, Christian-Albrechts-Universitaet zu Kiel

LCModel (Version 6.3-1J) Copyright: S.W. Provencher. *Ref.: Magn. Reson. Med. 30:672-679 (1993).*

19-June-2019 17:28

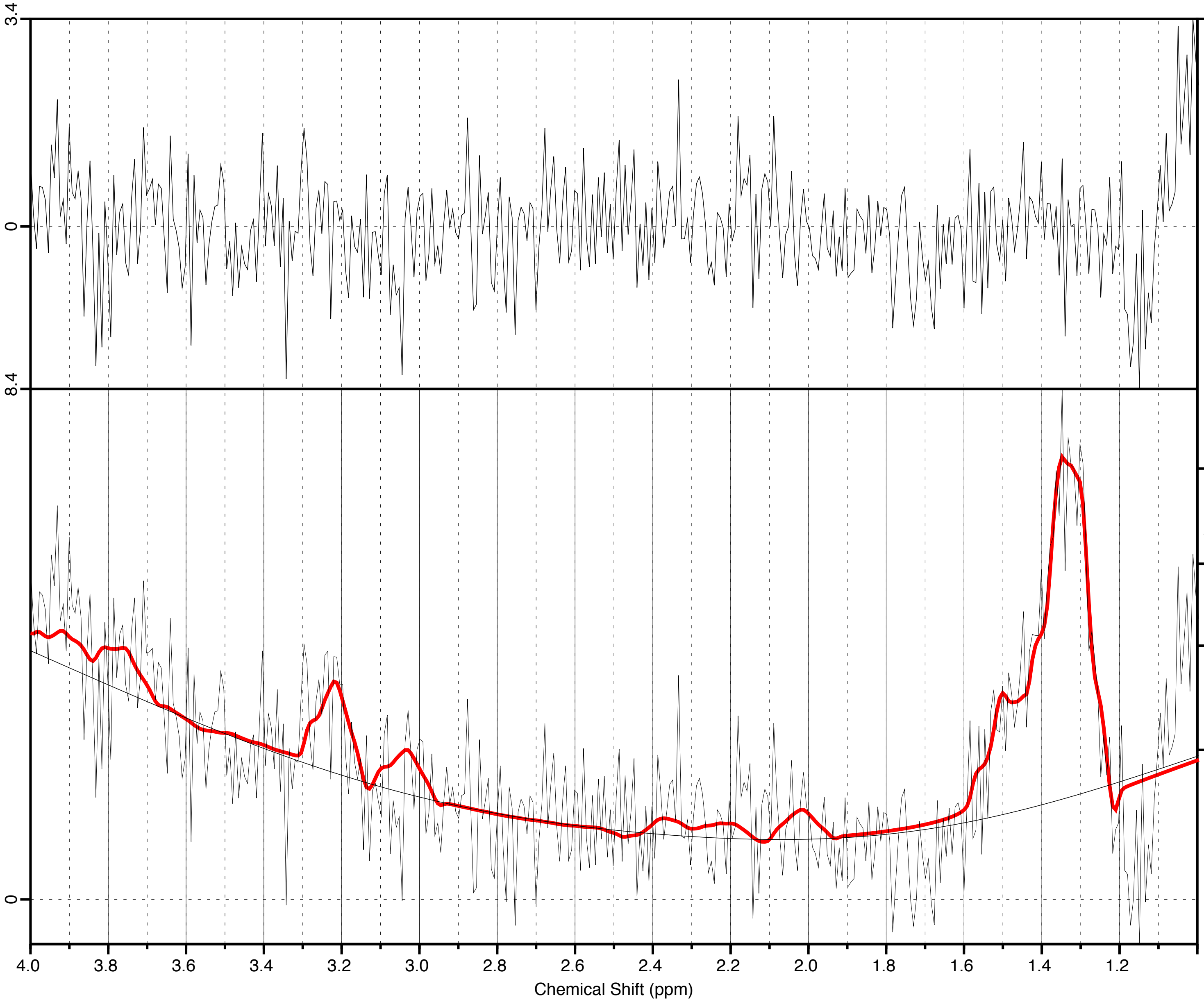

Conc.	%SD	/GPC+PC	Metabolite
127.586	20%	5.611	Ala
33.349	45%	1.467	Cr
0.000	999%	0.000	PCr
63.210	71%	2.780	Gln
0.000	999%	0.000	Glu
0.000	999%	0.000	GPC
22.740	25%	1.000	PCh
0.000	999%	0.000	GSH
34.288	453%	1.508	Ins
**345.591**	**7%**	**15.198**	**Lac**
18.346	82%	0.807	NAA
2.875	95%	0.126	NAAG
1.197	119%	5.3E-02	Scyllo
6.685	106%	0.294	Tau
0.000	999%	0.000	-CrCH2
22.740	25%	1.000	GPC+PCh
21.221	81%	0.933	NAA+NAAG
33.349	45%	1.467	Cr+PCr
63.210	71%	2.780	Glu+Gln
0.000	999%	0.000	Lip13a
0.000	999%	0.000	Lip13b
2.27E-05	999%	1.0E-06	Lip20
0.000	999%	0.000	Lip13a+Lip13b

DIAGNOSTICS

1 info TWOREG 8
1 info FINOUT 9

MISCELLANEOUS OUTPUT

FWHM = 0.084 ppm S/N = 3
Data shift = 0.015 ppm
Ph: -26 deg 5.6 deg/ppm

INPUT CHANGES

```
deltat= 5.000e-04
echot= 288.00
filbas= '/home/stu111733/.lcmodel/
  basis-sets/gamma_press_te288_128
  mhz_662d.basis'
hzpppm= 1.2776e+02
icolen= 7
icolst= 5
irowen= 12
irowst= 9
islice= 1
```

P44 - tumor
voxel 11;5

Row#11 Col#5 MR Hirn (2018.11.14 09:25:43) Subject 14 8.00e-01 mL, TE/TR/NS=288/1500/1 (s2DSI_TE288 TR1500)

Data of: Sektion Biomedizinische Bildgebung, Christian-Albrechts-Universitaet zu Kiel

LCModel (Version 6.3-1J) Copyright: S.W. Provencher. Ref.: Magn. Reson. Med. 30:672-679 (1993).

19-June-2019 17:28

Conc.	%SD	/GPC+PC	Metabolite
127.586	20%	5.611	Ala
33.349	45%	1.467	Cr
0.000	999%	0.000	PCr
63.210	71%	2.780	Gln
0.000	999%	0.000	Glu
0.000	999%	0.000	GPC
22.740	25%	1.000	PCh
0.000	999%	0.000	GSH
34.288	453%	1.508	Ins
**345.591**	**7%**	**15.198**	**Lac**
18.346	82%	0.807	NAA
2.875	95%	0.126	NAAG
1.197	119%	5.3E-02	Scyllo
6.685	106%	0.294	Tau
0.000	999%	0.000	-CrCH2
22.740	25%	1.000	GPC+PCh
21.221	81%	0.933	NAA+NAAG
33.349	45%	1.467	Cr+PCr
63.210	71%	2.780	Glu+Gln
0.000	999%	0.000	Lip13a
0.000	999%	0.000	Lip13b
2.27E-05	999%	1.0E-06	Lip20
0.000	999%	0.000	Lip13a+Lip13b

DIAGNOSTICS

1 info TWOREG 8
1 info FINOUT 9

MISCELLANEOUS OUTPUT

FWHM = 0.084 ppm S/N = 3
Data shift = 0.015 ppm
Ph: -26 deg 5.6 deg/ppm

INPUT CHANGES

deltat= 5.000e-04
echot= 288.00
filbas= '/home/stu111733/.lcmodel/basis-sets/gam
ma_press_te288_128mhz_662d.basis'
hzpppm= 1.2776e+02
icolen= 7
icolst= 5
irowen= 12
irowst= 9
islice= 1
lps= 8
ltable= 7
ndcols= 16
ndrows= 20
ndslic= 1
nunfil= 1024
ppmend= 1.0
ppmst= 4.0
savdir= '/home/data/Studies/GBM_Spektro/
results/s14/'
sptype= 'tumor'
srcraw= '/home/data/Studies/GBM_Spektro/data/
s14/01/spectro01/spectro01_8_1_raw_act .SDAT'

P44 - edema
voxel 8;5

Row#8 Col#5 MR Hirn (2018.11.14 09:25:43) Subject 14 8.00e-01 mL, TE/TR/NS=288/1500/1 (s2DSI_TE288 TR1500)

Data of: Sektion Biomedizinische Bildgebung, Christian-Albrechts-Universitaet zu Kiel

LCModel (Version 6.3-1J) Copyright: S.W. Provencher.   *Ref.: Magn. Reson. Med. 30:672-679 (1993).*   *28-November-2019 14:47*

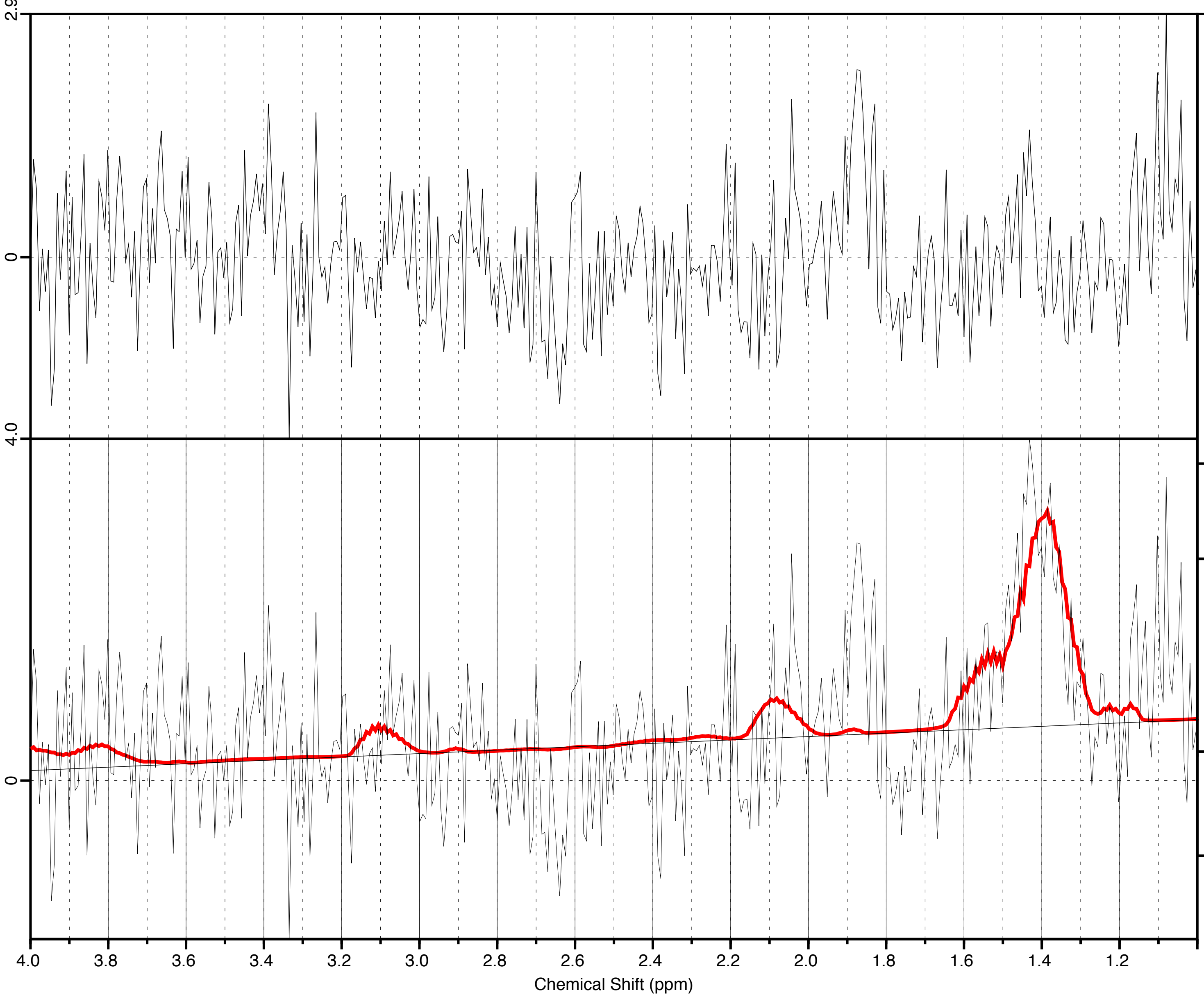

Conc.	%SD	/GPC+PC	Metabolite
64.102	35%	0.000	Ala
0.000	999%	0.000	Cr
18.465	80%	0.000	PCr
13.452	305%	0.000	Gln
0.000	999%	0.000	Glu
0.000	999%	0.000	GPC
0.000	999%	0.000	PCh
0.000	999%	0.000	GSH
0.000	999%	0.000	Ins
161.596	16%	0.000	Lac
22.447	61%	0.000	NAA
3.367	61%	0.000	NAAG
0.443	64%	0.000	Scyllo
2.213	64%	0.000	Tau
1.846	81%	0.000	-CrCH2
0.000	999%	0.000	GPC+PCh
25.814	61%	0.000	NAA+NAAG
18.465	80%	0.000	Cr+PCr
13.452	305%	0.000	Glu+Gln
0.000	999%	0.000	Lip13a
0.000	999%	0.000	Lip13b
0.000	999%	0.000	Lip20
0.000	999%	0.000	Lip13a+Lip13b

DIAGNOSTICS

**1 warning**	**STARTV 25**
1 info	STARTV 24
8 info's	RFALSI 11
5 info's	RFALSI 4
3 info's	TWOREG 8
1 info	TWOREG 9
1 info	FINOUT 9

MISCELLANEOUS OUTPUT

FWHM = 0.107 ppm   S/N =   2
Data shift =-0.099 ppm
Ph: -8 deg   14.8 deg/ppm

INPUT CHANGES

deltat= 5.000e-04
echot= 288.00
filbas= '/home/stu111733/.lcmodel/
  basis-sets/gamma_press_te288_128
  mhz_662d.basis'
hzpppm= 1.2776e+02

P44 - edema
voxel 8;5

Row#8 Col#5 MR Hirn (2018.11.14 09:25:43) Subject 14 8.00e-01 mL, TE/TR/NS=288/1500/1 (s2DSI_TE288 TR1500)

Data of: Sektion Biomedizinische Bildgebung, Christian-Albrechts-Universitaet zu Kiel

LCModel (Version 6.3-1J) Copyright: S.W. Provencher. *Ref.: Magn. Reson. Med. 30:672-679 (1993).*

28-November-2019 14:47

Conc.	%SD	/GPC+PC	Metabolite
64.102	35%	0.000	Ala
0.000	999%	0.000	Cr
18.465	80%	0.000	PCr
13.452	305%	0.000	Gln
0.000	999%	0.000	Glu
0.000	999%	0.000	GPC
0.000	999%	0.000	PCh
0.000	999%	0.000	GSH
0.000	999%	0.000	Ins
161.596	16%	0.000	Lac
22.447	61%	0.000	NAA
3.367	61%	0.000	NAAG
0.443	64%	0.000	Scyllo
2.213	64%	0.000	Tau
1.846	81%	0.000	-CrCH2
0.000	999%	0.000	GPC+PCh
25.814	61%	0.000	NAA+NAAG
18.465	80%	0.000	Cr+PCr
13.452	305%	0.000	Glu+Gln
0.000	999%	0.000	Lip13a
0.000	999%	0.000	Lip13b
0.000	999%	0.000	Lip20
0.000	999%	0.000	Lip13a+Lip13b

DIAGNOSTICS

```
1 warning   STARTV 25
1 info      STARTV 24
8 info's    RFALSI 11
5 info's    RFALSI  4
3 info's    TWOREG  8
1 info      TWOREG  9
1 info      FINOUT  9
```

MISCELLANEOUS OUTPUT

```
FWHM = 0.107 ppm    S/N =   2
Data shift =-0.099 ppm
Ph:  -8 deg      14.8 deg/ppm
```

INPUT CHANGES

```
deltat= 5.000e-04
echot= 288.00
filbas= '/home/stu111733/.lcmodel/basis-sets/gam
  ma_press_te288_128mhz_662d.basis'
```

```
hzpppm= 1.2776e+02
icolen= 7
icolst= 5
irowen= 8
irowst= 8
islice= 1
lps= 8
ltable= 7
ndcols= 16
ndrows= 20
ndslic= 1
nunfil= 1024
ppmend= 1.0
ppmst= 4.0
savdir= '/home/stu111733/.lcmodel/saved/MR_Hirn/
  s14/2018.11.14_09:25:43/'  sptype= 'tumor'
srcraw= '/home/data/Studies/GBM_Spektro/data/s14

  /spectro01/spectro01_8_1_raw_act.SD    AT'
```
